# Mapping transcription factor binding sites by learning UV damage fingerprints

**DOI:** 10.1093/nar/gkaf1014

**Published:** 2025-10-14

**Authors:** Hannah E Wilson, Scott Stevison, Levi Lamprey, John J Wyrick

**Affiliations:** School of Molecular Biosciences, Washington State University, Pullman, WA 99164, USA; School of Molecular Biosciences, Washington State University, Pullman, WA 99164, USA; School of Molecular Biosciences, Washington State University, Pullman, WA 99164, USA; School of Molecular Biosciences, Washington State University, Pullman, WA 99164, USA

## Abstract

Deciphering transcriptional networks requires methods to accurately map binding sites of sequence-specific transcription factors (ssTFs) across the genome. Here, we show that ssTF binding induces distinct patterns of UV-induced cyclobutane pyrimidine dimers (CPDs), and that these CPD ‘fingerprints’ can be exploited by machine learning methods to identify ssTF binding sites (TFBS). As a proof of principle, we analyzed CPD-seq data from yeast cells using the Random Forest algorithm to identify 75 TFBS bound by the Hap2/Hap3/Hap5 ssTF complex, including ∼25 new sites missed by previous chromatin immunoprecipitation (ChIP)-based experiments. Parallel analysis of the Gcr1 ssTF using a neural network trained on CPD-seq data including only 6 known binding sites identified 63 Gcr1 TFBS across the genome. Our analysis indicates that the newly identified TFBS are associated with many genes that function in expected categories (e.g. mitochondrial respiration or glycolysis), and whose mRNA levels are down-regulated in ssTF mutants. Similar analysis of CPD-capture-sequencing data from human cells identified new sites bound by the homologous Nuclear Factor-Y complex. These findings indicate that distinct cellular patterns of UV damage occurring at different classes of TFBS can be recognized by machine learning methods to map these regulatory elements with improved accuracy and single-nucleotide resolution.

## Graphical Abstract

**Graphical Abstract:**
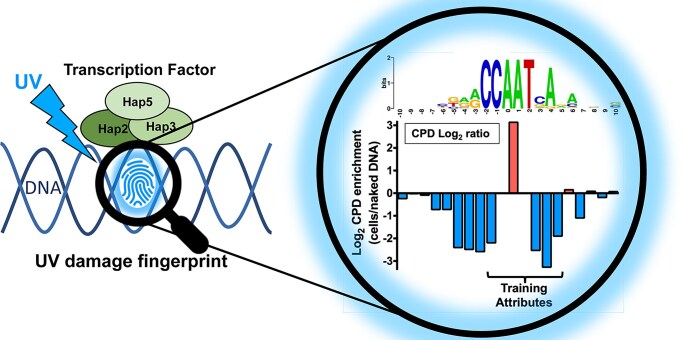

## Introduction

Precise regulation of gene transcription plays a critical role in enabling cells to grow, differentiate, and respond appropriately to changes in their environment. While a number of mechanisms, including chromatin accessibility and epigenetic modifications, contribute to transcriptional regulation, the primary regulatory mechanism involves sequence-specific transcription factor (ssTF) proteins [1, 2]. ssTFs bind to short DNA sequence motifs, ∼5–15 base pairs (bp) in length, which are typically located in promoter sequences immediately upstream of genes or in more distal enhancer regions [3]. Once bound to its cognate binding sequence, a ssTF activates or represses the transcription of neighboring genes, often by recruiting (or inhibiting the recruitment of) the RNA polymerase II machinery [1, 2]. For example, the yeast Hap2/Hap3/Hap5 ssTF complex binds to CCAAT sequence motifs in promoter regions to activate (in conjunction with Hap4) the expression of heme- and oxygen-responsive genes [4, 5]. These include many genes involved in heme synthesis, the mitochondrial electron transport chain, and other facets of aerobic respiration. A homologous human ssTF complex known as Nuclear Factor-Y (NF-Y) binds to the same sequence motif in the promoters of many human genes to regulate their transcription [6]. To understand the function of these and other ssTFs (e.g. Gcr1, a key regulator of glycolysis genes [7]), it is important to accurately map the DNA sites to which they bind in living cells.

Much of our current knowledge about ssTF binding sites (TFBS) is derived from chromatin immunoprecipitation (ChIP) experiments [8] and related methods (e.g. CUT&RUN, etc., [9]). ChIP-based methods typically employ formaldehyde crosslinking to capture protein-DNA interactions in living cells, followed by immunoprecipitation with specific antibodies to isolate DNA crosslinked to a protein of interest. Interrogation of the resulting ChIP DNA, originally by microarrays (ChIP-chip), and subsequently by next-generation sequencing (ChIP-seq), provides a genome-wide survey of DNA sites bound by a particular ssTF [8, 10]. The highest-resolution version of these methods, known as ChIP-exonuclease (ChIP-exo), employs lambda exonuclease to digest the immunoprecipitated DNA, and thereby precisely maps the crosslink position in the DNA fragment [11]. However, ChIP-based methods have been reported to have systematic biases [12–14], require highly specific (i.e. “ChIP-grade”) antibodies, and typically can only measure the binding profile of a single protein at a time, highlighting the need for alternative methods to map cellular TFBS.

TFBS have also been characterized by an orthogonal approach known as DNA footprinting [15, 16]. Footprinting methods typically employ a DNA cleaving enzyme (e.g. DNase I) or DNA damaging agent such as dimethyl sulfate (DMS) or UV light [15, 17–20]. In footprinting experiments, protein binding to DNA significantly suppresses DNase I cleavage or the formation of alkylation damage, resulting in a ‘footprint’ in the pattern of damage induced [17–20]. However, UV damage is unusual in that ssTF binding not only suppresses but also can induce UV-induced CPD formation, resulting in a unique CPD “fingerprint.” These UV damage fingerprints are caused by changes in the conformation of the DNA site upon ssTF binding, which render the DNA more (or less) susceptible to CPD formation [21, 22]. We and others have shown that next-generation sequencing methods such as CPD-sequencing (CPD-seq) can detect these CPD fingerprints associated with DNA-bound TFs in UV-irradiated cells [22–27]. However, whether such methods can be used to map individual ssTF binding sites across the genome is unclear.

Here, we use CPD-seq to map UV damage at unprecedented sequencing depth across the genome of UV-irradiated cells and naked DNA controls. We use a machine learning algorithm known as Random Forest [28] to identify a CPD fingerprint associated with DNA-binding by the yeast Hap2/Hap3/Hap5 (Hap2/3/5) complex, and use this algorithm to identify Hap2/3/5 binding sites across the yeast genome and binding sites of the homologous NF-Y complex in human promoter and enhancer regions. We further show that a different machine learning algorithm known as neural networks (NN) can also use CPD-seq data to identify binding sites of a different ssTF known as Gcr1, despite there being only a few known TFBS available for training. These data indicate that “CPD fingerprinting” can be used to simultaneously identify binding sites for multiple ssTFs at single-nucleotide resolution across the genome in living cells.

## Materials and methods

### CPD-seq library preparation

CPD-seq of UV-irradiated yeast cells or isolated yeast genomic DNA was performed essentially as previously described [29]. Briefly, yeast cells were grown to mid-log phase and UV irradiated, followed by harvesting by centrifugation and genomic DNA isolation (see Supplemental Materials and Methods for details). As a naked DNA control, isolated yeast genomic DNA was UV-irradiated on ice on coverslips *in vitro*. The purified genomic DNA from UV-irradiated yeast cells or genomic DNA was subsequently sonicated (Diagenode Biorupter 3000; 25 cycles, 30 s ON/OFF) to produce ∼200–600 bp fragments. First adapter sequences were ligated to both ends of the DNA fragments and any remaining free 3′ hydroxyls were blocked via reaction with terminal transferase (New England Biolabs; NEB) and dideoxy ATP. T4 endonuclease V (NEB) and APE1 (NEB) were then used to specifically cleave just upstream each CPD lesion. A biotinylated second adapter was then ligated to the free 3′ hydroxyl left by T4 endonuclease V and APE1 cleavage. Fragments ligated to the second adapter were pulled down using streptavidin beads (Thermo Fisher Scientific). Fragments were amplified via low-cycle PCR and sequenced via Illumina sequencing. Ampure XP beads (Cytiva) were employed for both fragment size selection and clean-ups following each enzymatic step.

### CPD-seq analysis

CPD-seq analysis was performed essentially as previously described [30], with slight modifications detailed below. CPD-seq reads were trimmed using Trimmomatic v0.39 (with arguments: ILLUMINACLIP:[sequencing_reads_file]:2:30:10, [31]) or bbduk (with arguments: ktrim = r k = 23 mink = 16 hdist = 2 tpe tbo) and then aligned to the yeast genome (saccer3) using Bowtie2 software [32]. The resulting SAM file was converted to a BAM file using SAMtools [33], and the BAM file was converted to a BED file using BEDTools [34]. A custom Perl script was used to identify the CPD-forming dinucleotide sequence on the opposite strand immediately upstream of the 5′ end of the sequencing read, and only CPD-seq reads mapping to a lesion-forming dipyrimidine sequence (i.e. TT, TC, CT, or CC) were analyzed further. The BED files were split into separate plus and minus strand files and converted into wig files using custom Perl scripts or IGVtools [35]. The lesion was assigned to a half-integer position corresponding to the average of the positions of the DNA bases forming the CPD (i.e. a CPD forming between positions 10 and 11 was given the position of 10.5). We also calculated the CPD induction values, which consisted of the difference in CPD counts at each position in the cellular DNA sample relative to the normalized naked DNA control. Average induction values were calculated by dividing the total induction value by the number of lesion-forming dipyrimidine positions at that location in the motif. To visualize CPD induction values and counts in IGV [35], the fractional/decimal was eliminated (i.e. a CPD at position 10.5 was assigned to position 10), essentially assigning the CPD count to the left (i.e. smaller) position in the dipyrimidine sequence. CPD counts were normalized based on the total number of CPD-seq reads at dipyrimidine sequences in each sample. Visualization of CPD induction values was performed using two replicates of a UV-irradiated cellular sample and a matched UV-irradiated naked DNA control. When analyzing CPD induction patterns in aggregate for TFBS or performing machine learning analysis, these replicates were combined and an additional sequencing library derived from resequencing one of the cellular replicates to obtain additional sequencing depth was also included. Analysis of CPD induction at yeast TFBS for all 78 transcription factors characterized by ChIP-exo [36] was performed using custom Perl scripts, essentially as previously described [37]. Uniform manifold approximation and projection (UMAP) analysis was performed using the umap package for R software for average CPD induction values (i.e. averaged by the number of binding sites analyzed) between positions −5.5 and +5.5 relative to the motif center for each type of yeast TFBS. For transcription factor binding positions that occurred more than once in this data set, only the first instance for each ssTF was analyzed.

### Human CPD-seq and CPD-capture-seq data analysis

Average CPD induction was calculated for previously published CPD-seq data [21, 22] for UVC-irradiated TERT-immortalized normal human skin fibroblasts (NHF1) cells relative to normalized naked DNA controls (GEO data sets: GSE162125 and GSE103487). Average CPD induction was characterized at NF-YA/B binding sites identified by ChIP-seq data sets from ENCODE [38, 39] and located in a melanocyte DNase I hypersensitivity region [40], as previously described [22]. For analysis of average CPD induction associated with capture regions, we analyzed published CPD-capture-seq data [41] for UVC- and predominately UVB-irradiated NHF1 cells relative to matched naked DNA controls (GEO data set: GSE225362) at NF-Y binding sites located within a 360 bp window centered in the middle of capture region.

### Random forest analysis of CPD-seq data

The Waikato Environment for Knowledge Analysis (Weka) RandomForest tool [42] was used to identify Hap2/3/5 binding sites based on patterns in UV damage induction. As attributes, we analyzed CPD induction values and log_2_ ratios of CPD counts in UV-irradiated WT yeast cells relative to the normalized naked DNA controls. For the log_2_ ratios, the data was floored to a minimum CPD count of 10 at each position. We determined the CPD induction and floored log_2_ ratio positions −1.5, +0.5, +2.5, +3.5, and + 4.5, with position “0” correlating to the central adenine in the CCAAT binding motif. These attributes were chosen in part by information provided by Weka's “InfoGainAttributeEval” attribute evaluator tool. The training data consisted of 35 Hap2/3/5 binding sites previously identified by ChIP-exo [36] and 7000 negative instances comprised of CCAAT sequences occurring inside of yeast open reading frames (ORFs) and located at least 100 bp from the start or end of the ORF. The Random Forest algorithm was run with 100 iterations. The trained model was then re-evaluated on a test set of all 33 720 CCAAT sequences present across the yeast genome (excluding ribosomal DNA regions) to identify Hap2/3/5 binding sites. The Random Forest algorithm trained on the yeast data was used to predict NF-Y bound CCAAT sites located in the central 360 bp of a capture region using the same parameters.

### Neural network analysis of CPD-seq data

The Weka Multilayer Perceptron (i.e. NN) tool was used to identify Gcr1 binding sites based on average CPD induction values and log_2_ ratios of CPD counts in UV-irradiated WT cells relative to the normalize naked DNA controls, as described above. The CPD induction values and floored log_2_ ratios were determined for positions −1.5, -0.5, +0.5, and + 1.5, with position “0” corresponding to the central cytosine in the CTTCC binding motif. These attributes were chosen in part by information provided by Weka's “InfoGainAttributeEval” attribute evaluator tool. The training data consisted of six Gcr1 binding sites previously identified by ChIP-exo [36] and 7000 negative instances comprised of [C/A]TTCC sequences occurring within yeast ORFs at least 100 bp from the start or end of the ORF. The trained model was then re-evaluated on a test set of all 56 312 [C/A]TTCC sequences present across the yeast genome (excluding ribosomal DNA regions) to identify Gcr1 binding sites. The multilayer perceptron algorithm was also trained on the Hap2/3/5 training data, in order to predict Hap2/3/5 binding sites, as described above for the Random Forest algorithm.

## Results

### CPD fingerprint associated with DNA-binding by the Hap2/Hap3/Hap5 complex in yeast

**Figure 1.**
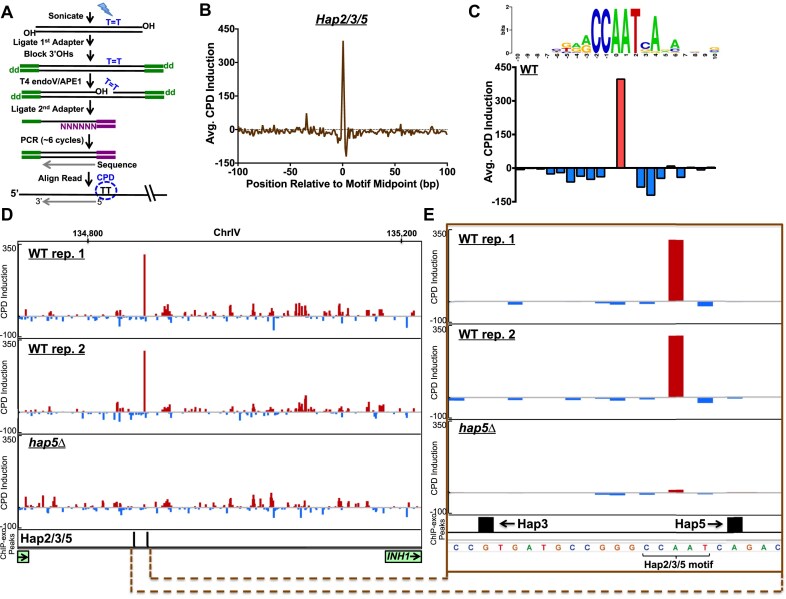
Using CPD-seq to analyze cellular UV damage patterns at Hap2/Hap3/Hap5 binding sites across the yeast genome. (**A**) Schematic detailing the experimental procedure for the cyclobutane pyrimidine dimer-sequencing (CPD-seq) method, which utilizes the repair enzymes T4 endonuclease V (T4 endoV) and apurinic/apyrimidinic endonuclease 1 (APE1) to map UV-induced CPDs across the genome at single- nucleotide resolution. (**B**) Plot showing average CPD induction in UV-irradiated wild-type (WT) yeast cells relative to UV-irradiated naked DNA controls at DNA regions adjacent to 35 known Hap2/Hap3/Hap5 binding sites identified by ChIP-exo. Average CPD induction is defined as the difference in CPD counts between UV-irradiated cells and the normalized naked DNA control, divided by the total number of DNA positions (i.e. binding sites) analyzed. (**C**) Close-up showing the average CPD induction within the Hap2/Hap3/Hap5 binding motif for 35 known binding sites in WT yeast cells. CPD induction is calculated for half-integer positions (i.e. average CPD induction at position −1.5 corresponds to CPDs forming between CC bases at positions −1 and −2 in the binding site), as before. DNA sequence logo was generated using the weblogo software. (**D**) Snapshot of CPD induction in the promoter region of the INHibitor (of F1F0-ATPase) *INH1* gene (indicated by green rectangle on the bottom right of the display), which regulates the activity of the mitochondrial F1F0-ATPase. CPD induction is depicted for two independent CPD-seq experiments in which UV-irradiated WT cells were each compared to a naked DNA control. The third track shows cumulative CPD induction in two control CPD-seq data sets for *hap5*Δ cells relative to the naked DNA controls. The bottom track shows aggregate positions of ChIP-exo peaks derived from Hap2, Hap3, and Hap5 ChIP-exo experiments. Image generated using the integrated genomics viewer (IGV) software. (**E**) Close-up of CPD induction and ChIP-exo peaks at the Hap2/Hap3/Hap5 binding site in the *INH1* promoter.

To map TFBS across the genome using UV damage fingerprinting, we mapped UV-induced CPD lesions (see Supplementary Table S1) at unprecedented sequencing depth using CPD-seq [23]. CPD-seq uses the DNA repair enzymes T4 endonuclease V and APE1 to map cyclobutane pyrimidine dimers (CPDs) in UV-irradiated cells across the genome at single-nucleotide resolution (Fig. 1A). We performed CPD-seq on replicate WT yeast cell cultures that were harvested immediately (i.e. 0 h [hr]) after UV-irradiation. The resulting CPD-seq libraries were sequenced at ∼29-fold greater sequencing depth than previous libraries, enabling us to accurately measure damage formation at individual TFBS (Supplementary Fig. S1A,B). As a control, we also UV-irradiated replicate samples of purified yeast genomic DNA *in vitro*, in order to control for effects of DNA sequence composition on UV damage formation [23]. After normalizing the two sets of samples, we determined the level of CPD induction in UV-irradiated cells relative to the naked DNA control, which we defined as simply the difference in normalized CPD counts between the cellular and naked DNA samples (Supplementary Fig. S2).

Analysis of CPD induction at 35 previously identified Hap2/3/5 binding sites identified by ChIP-exo [36] revealed significantly higher CPD levels in bound motifs in UV-irradiated cells (Fig. 1B). Closer inspection indicated that average CPD induction is specifically elevated at the AA position in the CCAAT motif (Fig. 1C), due to UV damage forming on the TT dinucleotide on the opposite strand (i.e. ATTGG). These findings indicate that binding by the Hap2/3/5 complex in cells stimulates CPD formation at this TT dinucleotide, consistent with our previous report [37]. In contrast, flanking positions in the Hap2/3/5 binding motif show a negative CPD induction (Fig. 1B and C), indicating that Hap2/3/5 binding suppresses CPD formation at these positions in UV-irradiated cells relative to the unbound naked DNA control.

To confirm that this CPD fingerprint is caused by Hap2/3/5 binding, we performed CPD-seq on UV-irradiated yeast cells in which the gene encoding the Hap5 subunit, which is required for Hap2/3/5 complex integrity and DNA-binding activity [43], was deleted (i.e. *hap5*Δ). Again, we normalized replicate cellular CPD-seq data from *hap5*Δ cells, and calculated the average CPD induction relative to the naked DNA control. The results indicated that *hap5*Δ eliminated CPD modulation at Hap2/3/5 binding sites (Supplementary Fig. S3A and B), confirming that UV damage modulation at these sites is a consequence of Hap2/3/5 complex binding.

Closer inspection of CPD induction patterns in the promoters of individual genes indicated that there were discernible peaks of CPD induction associated with Hap2/3/5 binding sites. For example, analysis of the *INH1* gene, which regulates the mitochondrial ATP synthase complex [44], revealed a peak of CPD induction in each of the replicate WT experiments at a position −308 bp upstream of the *INH1* coding region (Fig. 1D). The peak of CPD induction is located at the AA/TT dinucleotide sequence in a Hap2/3/5 motif, in close proximity to called ChIP-exo peaks for the Hap3 and Hap5 subunits (Fig. 1E). Importantly, CPD induction at the site is lost in the *hap5*Δ mutant (Fig. 1D and E). Taken together, these findings indicate that CPD-seq maps can reveal UV damage patterns associated with ssTF binding in the promoters of individual yeast genes.

### Mapping Hap2/3/5 binding sites based on their CPD fingerprint using the Random Forest algorithm

**Figure 2.**
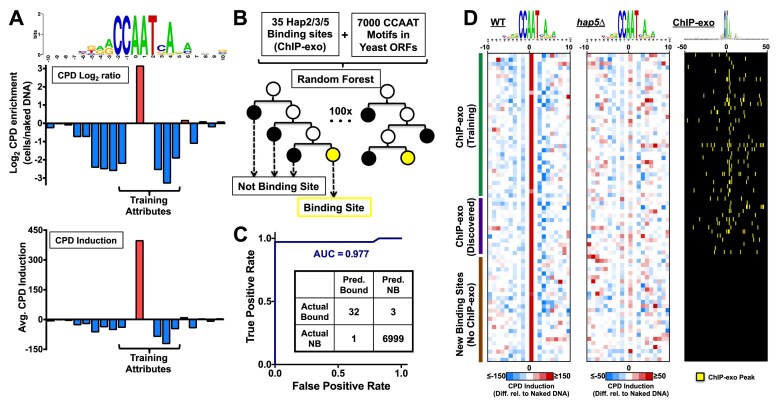
Using the Random Forest machine learning method to identify Hap2/3/5 binding sites based on their CPD fingerprint across the yeast genome. (**A**) Graph depicting training attributes for the 35 known Hap2/3/5 binding sites. Bottom panel is average CPD induction (same as Fig. 1C). Middle panel is log_2_ ratio of CPD enrichment in UV-irradiated WT cells relative to the naked DNA control, after flooring data to a minimum CPD count of 10. Top panel is the sequence logo of the analyzed binding sites (same as Fig. 1C). Only data for positions −1.5 to + 4.5 for CPD induction and CPD log ratio were used as training attributes. (**B**) Schematic showing machine learning procedure using Random Forest. 35 known Hap2/3/5 binding sites were used as positive examples and 7000 CCAAT motifs inside yeast ORFs were used as negative examples. The attribute data from each of these examples were used to train Random Forest implemented in the Weka software [42] to identify Hap2/3/5 binding sites. (**C**) Receiver operating characteristic (ROC) curve for Random Forest model trained on 35 positive examples and 7000 negative examples using five-fold cross validation. The area under the curve (AUC) value of the ROC curve is indicated. Inset shows the confusion matrix from the five-fold cross validation analysis, with “actual bound” indicating the 35 positive examples and “actual not bound” (NB) indicating the 7000 negative examples, while “predicted” (Pred.) indicates the number of bound and not bound identified sites by the Random Forest algorithm. (**D**) Plot showing CPD induction values in a 20 bp window surrounding the center of the 75 Hap2/3/5 binding sites identified by the Random Forest algorithm. Each row depicts the CPD induction values of a single binding site. Rows are organized with the 35 binding sites in the training data on top (green rectangle), 14 identified binding sites associated with a nearby ChIP-exo peak, but not present in the training data (i.e. “ChIP-exo discovered,” purple rectangle) in the middle, and 26 binding sites not associated with a Chip-exo peak (i.e. “new binding sites, no ChIP-exo,” brown rectangle) at the bottom. Columns indicate the positions of damage induction relative to the center of the bound motif. Colors indicate the sign and magnitude of the damage induction, with red indicating higher CPD induction in cells relative to naked DNA, and blue indicating lower CPD induction in cells relative to the naked DNA control (see color bar). Top of the panel gives the sequence logo for the 75 identified binding sites, generated using the weblogo software [63]. Left panel indicates CPD induction data for WT cells, middle panel depicts CPD induction data for *hap5*Δ cells, and right panel indicates the positions of ChIP-exo peaks within 50 bp of the 75 identified binding sites, derived from the aggregate of published ChIP-exo data for Hap2, Hap3, and Hap5 [36].

Since previous studies have indicated that machine learning methods can be a powerful way of identifying TFBS and analyzing their regulatory logic [45, 46], we wondered whether we could use machine learning to identify Hap2/3/5 binding sites across the yeast genome based on their CPD fingerprint. We initially chose to use a Random Forest (RF) algorithm, since this decision tree-based method has been shown to be a simple, fast, and robust solution for a variety of different machine learning tasks [28, 47], and because the discrete, position-specific nature of the CPD-seq data at TFBS lends itself well to RF classification. We calculated CPD induction at the hotspot associated with the AA/TT dinucleotide at position 0/+1 in the Hap2/3/5 binding motif (i.e. position + 0.5), as well as values at neighboring positions (−1.5, +2.5, +3.5, and + 4.5) where CPD induction is suppressed. In parallel, we also calculated the log_2_ ratio of normalized CPDs in the cellular samples relative to the naked DNA control (after flooring the data to a minimum of 10 CPD-seq reads, see Materials and Methods) at each of the same positions in the Hap2/3/5 binding motif (i.e. −1.5, +0.5, +2.5, +3.5, and + 4.5), giving a total of 10 attributes associated with each potential CCAAT motif (Fig. 2A).

We trained the RF classifier on these attributes for 35 positive examples, derived from published Hap2/3/5 ChIP-exo data [36], and 7000 negative examples, consisting of CCAAT motif instances present inside of yeast ORFs (Fig. 2B). These latter binding sites were used as negative (i.e. unbound) examples because intragenic motif instances are unlikely to be bound. Five-fold cross validation indicated that the trained RF classifier successfully predicted Hap2/3/5 binding with single-nucleotide resolution at > 90% of the positive examples and only had a single instance of a false positive prediction for the negative examples (i.e. ∼99.99% specificity; Fig. 2C). Analysis of the receiver operating characteristic (ROC) curve gave an area under the curve (AUC) value of 0.977 (Fig. 2C), indicating that the RF classifier performed very well on the training data with five-fold cross validation.

The trained RF classifier was then used to identify bound Hap2/3/5 sites throughout the yeast genome using the WT CPD-seq data. This analysis identified 75 bound Hap2/3/5 sites out of a total of 33 720 potential binding sites (i.e. CCAAT motifs) in the yeast genome (Fig. 2D). These 75 identified binding sites included all 35 of the training examples, as well as 14 additional binding sites that were associated with a Hap2, Hap3, or Hap5 ChIP-exo peak located within 50 bp of the identified binding site (ChIP-exo “Discovered” in Fig. 2D). Moreover, 26 new binding sites were discovered that were not associated with a nearby Hap2, Hap3, or Hap5 ChIP-exo peak (“New Binding Sites” in Fig. 2D). All 75 identified binding sites showed a clear Hap2/3/5 CPD fingerprint, consisting of elevated CPD levels at position +0.5, reflecting CPD formation at the central AA/TT dinucleotide (i.e. positions 0 and + 1) in the binding motif, and generally decreased CPD levels at flanking positions (e.g. positions +2.5, +3.5, etc., see Fig. 2D). These patterns of CPD induction were essentially absent in the *hap5*Δ mutant (Fig. 2D), indicating that they were a consequence of Hap2/3/5 binding.

**Figure 3.**
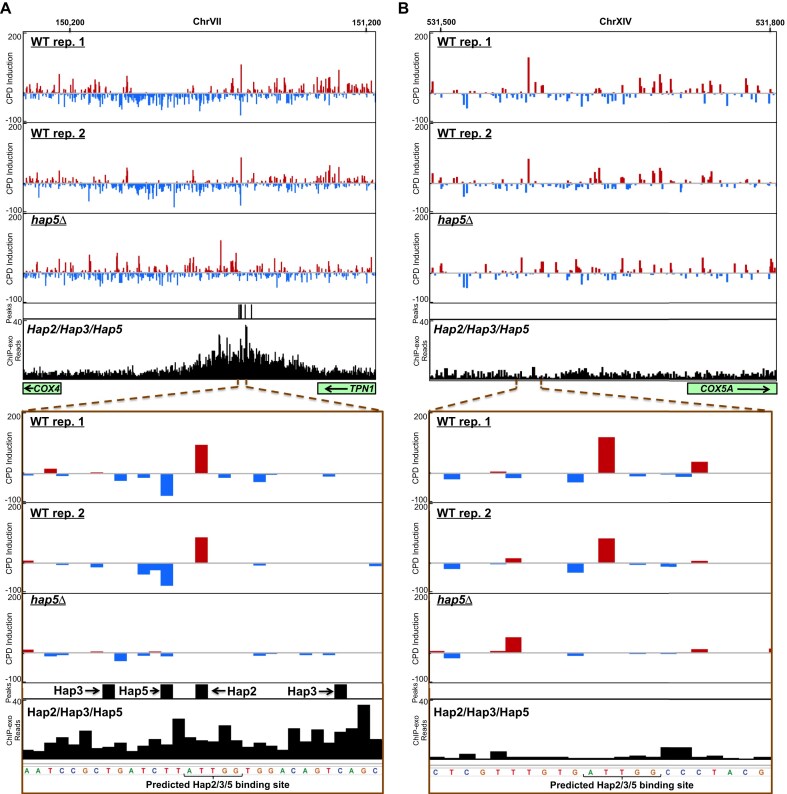
(**A**) Visualization of CPD induction and ChIP-exo data near a predicted Hap2/3/5 binding site in promoter of the *COX4* gene. Top panel shows CPD induction in derived from normalized CPD-seq data for two wildtype (WT) replicates and *hap5*Δ mutant cells, relative to naked DNA controls. The positions of ChIP-exo peaks and ChIP-exo reads for the Hap2, Hap3, and Hap5 subunits in aggregate are also depicted. Position of *COX4* gene is indicated with green rectangle on bottom left of panel. Bottom panel shows a zoomed in view near the predicted Hap2/3/5 binding site. ChIP-exo data from [36]. (**B**) Same as panel A, except for a predicted Hap2/3/5 binding site in the promoter of the *COX5A* gene. Figure generated using IGV [35].

One of the identified Hap2/3/5 binding sites was associated with the *COX4* gene, which encodes a subunit of the mitochondrial Cytochrome c oxidase complex [4]. This binding site is associated with ChIP-exo peaks for each of the Hap2/3/5 complex subunits, which cluster around the identified Hap2/3/5 binding site (Fig. 3A). A second identified binding site was associated with the *COX5A* gene, which also encodes a Cytochrome c oxidase subunit. Unlike *COX4*, there were no peaks of Hap2, Hap3, or Hap5 binding associated with the identified binding site in the *COX5A* promoter, nor was there enrichment of ChIP-exo sequencing reads for these subunits in this region (Fig. 3B). Analysis of published ChIP-chip data for Hap2, Hap3, and Hap5 [48] indicated that *COX5A* was also not identified as a Hap2/3/5 binding target by ChIP-chip assays. Similar analysis of the other 25 newly discovered Hap2/3/5 binding sites indicated that only two of these targets (*ATP1* and *YGR121W-A*) were identified by prior ChIP-chip analysis of Hap2, Hap3, or Hap5 binding [48, 49]. Previous studies identified both *COX4* and *COX5A* as target genes directly regulated by the Hap2/3/5 complex (reviewed in [4]). However, published ChIP-exo (and ChIP-chip) data [36, 48] only identified *COX4* as a bound target gene, while RF analysis of our CPD-seq data clearly indicates that both *COX4* and *COX5A* are bound by the Hap2/3/5 complex. Other previous identified Hap2/3/5 target genes, including *COR1* and *QCR8*, which encode subunits of the Ubiquinol cytochrome c reductase complex [4], were also identified by RF analysis of our CPD-seq data, but were missed by ChIP-based methods. These findings suggest that the RF algorithm can identify Hap2/3/5 binding sites based on their CPD fingerprint, including sites consistently missed by ChIP-based methods.

### Function and regulation of new Hap2/3/5 target genes

**Figure 4.**
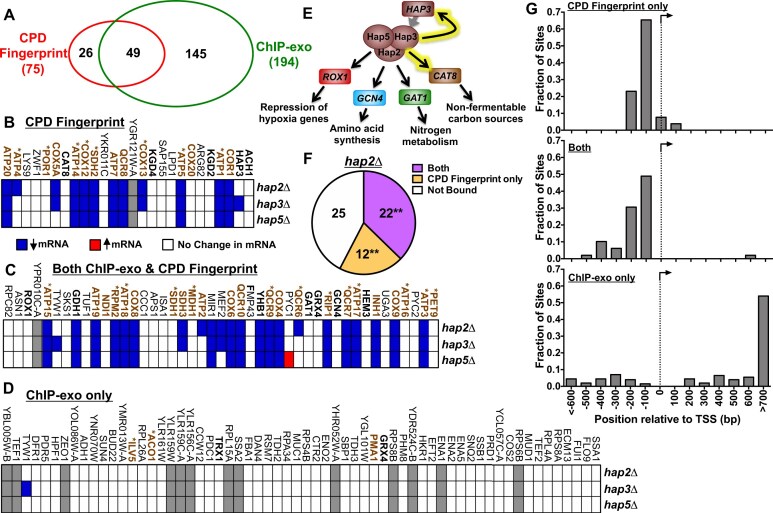
Characterization of Hap2/3/5 binding sites and associated target genes identified by CPD-seq. (**A**) Venn diagram showing overlap between Hap2/3/5 binding sites by Random Forest analysis of CPD-seq data (i.e. CPD fingerprint) and peaks/binding sites identified by ChIP-exo [36]. Binding site is located within 50 bp of a ChIP-exo peak were considered to be overlapping. (*B*-*D*) Lists of Hap2/3/5 target genes identified by (**B**) CPD fingerprint only, (**C**) ChIP-exo and CPD fingerprint, and (**D**) ChIP-exo only. The names of genes that have an aerobic respiration deficiency when mutated are colored brown with an asterisk, while genes that function in the ‘electron transport and membrane-associated energy conservation’ functional category (but do not have an aerobic respiration phenotype) are colored brown but do not have an asterisk. Blue rectangles indicate genes whose mRNA levels that are significantly down-regulated in *hap2*Δ, *hap3*Δ, or *hap5*Δ mutants (*P* < 1 × 10^−6^, log_2_ ratio ≤ -0.5), red rectangles indicate genes whose mRNA levels that are significantly up-regulated in *hap2*Δ, *hap3*Δ, or *hap5*Δ mutants (*P* < 1 × 10^−6^, log_2_ ratio ≥ 0.5), white rectangles indicate no significant change in mRNA levels, and gray rectangles indicate no data is available for this gene. Gene expression data for *hap2*Δ, *hap3*Δ, or *hap5*Δ mutants is from [50]. (**E**) Schematic showing target genes encoding ssTFs that contain identified Hap2/3/5 binding sites identified by CPD fingerprinting. Rectangles indicate target genes, while circles indicate the Hap2/3/5 complex. Black arrows indicate regulatory interactions due to a bound Hap2/3/5 site in the promoter region of the gene. Black arrows glowing yellow indicate novel Hap2/3/5 targets identified by CPD fingerprinting but not ChIP-exo. (**F**) Pie chart indicating the number of down-regulated genes in *hap2*Δ mutant cells (*P* < 1 × 10^−6^, log_2_ ratio ≤ -0.5) that contain binding sites identified by both CPD fingerprint and ChIP-exo (‘Both’), CPD fingerprint only, or by neither method (‘Not Bound’). No down-regulated genes were identified by ChIP-exo only. **Indicates a significant overlap of Hap2/3/5 target genes with the genes down regulated in *hap2*Δ mutant cells; *P* < 0.0001, based on the hypergeometric distribution. (**G**) Fraction of Hap2/3/5 binding sites identified by CPD fingerprint (top panel), CPD fingerprint and ChIP-exo (‘Both’, middle panel), and ChIP-exo only (bottom panel) located at the indicated distance from the nearest TSS of a neighboring gene. TSS data is from [64].

Of the 75 Hap2/3/5 binding sites identified by CPD fingerprinting, 49 were within 50 bp of a ChIP-exo peak for Hap2, Hap3, or Hap5 (Fig. 4A). Additionally, there were 145 distinct ChIP-exo peaks for Hap2, Hap3, or Hap5 that were not associated with a binding site identified by CPD fingerprinting (Fig. 4A). To characterize the target genes associated with each of these sets of binding sites, we identified the gene with the transcription start site (TSS) closest to each binding site (see Materials and Methods). We then analyzed each of these genes using published mRNA expression data from yeast strains containing *hap2*Δ, *hap3*Δ, or *hap5*Δ mutants [50].

Of the 26 genes associated with newly discovered Hap2/3/5 binding sites (i.e. CPD fingerprinting only), 13 (50%) were significantly down-regulated in mRNA expression in one or more of the *hap2*Δ, *hap3*Δ, or *hap5*Δ mutant strains (Fig. 4B). Functional analysis using FunSpec [51] indicated that 13 of the 26 newly discovered target genes were associated with the ‘electron transport and membrane-associated energy conservation’ functional category (Fig. 4B), a statistically significant overlap (*P* < 1 × 10^−14^). Moreover, eight of the genes were reported to have a deficiency in aerobic respiration when mutated in yeast (i.e. gene names with asterisks in Fig. 4B, *P* = 2 × 10^−7^). This functional enrichment is consistent with the reported function of Hap2/3/5 in regulating heme-responsive genes involved in aerobic respiration and electron transport [4, 5]. Other notable targets including genes involved in the tricarboxylic acid (TCA) cycle (i.e. *KGD2*, *KGD4*), as well as the transcriptional regulators *CAT8* and *HAP3* (gene names highlighted in bold in Fig. 4B).

Similar analysis of the 49 target genes identified by both ChIP-exo and CPD fingerprinting again showed a significant fraction of genes (27 out of 49, 55%) whose mRNA expression is down-regulated in one or more of the *hap2*Δ, *hap3*Δ, or *hap5*Δ mutant strains (Fig. 4C). Gene function analysis again revealed a significant overlap of genes in the ‘electron transport and membrane-associated energy conservation’ functional category (Fig. 4C, *P* < 1 × 10^−14^). Moreover, 13 of the 49 target genes identified by both ChIP-exo and CPD fingerprinting were reported to have a deficiency in aerobic respiration when mutated in yeast (gene names with asterisks in Fig. 4C, *P* = 6.1 × 10^−11^), indicating these genes play a critical role in mitochondrial respiration.

In contrast, only 1 out of the 62 target genes identified by “ChIP-exo only” was significantly down-regulated in its mRNA levels in one or more of the *hap2*Δ, *hap3*Δ, or *hap5*Δ mutant strains (Fig. 4D). Moreover, gene function analysis did not reveal significant enrichment in the “electron transport and membrane-associated energy conservation” functional category (Fig. 4D, P > 0.05), nor was there a significant overlap of genes reported to have a deficiency in aerobic respiration when mutated in yeast (gene names with asterisks in Fig. 4D, *P* > 0.05).

Of the Hap2/3/5 target genes identified by CPD fingerprinting, five of these encode ssTFs (Fig. 4E). Three of these ssTFs (*GAT1*, *GCN4*, and *ROX1*) were identified as putative targets with varying degrees of evidence (see Supplementary Fig. S4) by previous ChIP-exo experiments [36], while the other two genes (*CAT8* and *HAP3*) are new targets identified by CPD-seq (Fig. 4E). *GAT1* and *GCN4* encode transcriptional activators that regulate the expression of genes involved in nitrogen metabolism and amino acid synthesis, respectively, while *ROX1* is a repressor of hypoxia genes under aerobic growth conditions [4]. This latter regulatory interaction is particularly noteworthy, since *ROX1* expression is known to be regulated by heme, and Rox1 functions in an integrated regulatory circuit with the transcriptional activators Hap1 and Hap2/3/5 to control the expression of heme- and oxygen-responsive genes [4, 5]. New targets identified by CPD-seq include a transcriptional regulator known as *CAT8*, which promotes the expression of genes involved in metabolism of non-fermentable carbon sources, and the *HAP3* gene, suggesting that *HAP3* expression is autoregulated (Fig. 4E).

Analysis of published gene expression data for *hap2*Δ mutant yeast [50], using a log_2_ ratio threshold of -0.5 and a stringent P-value cutoff of 1 × 10^−6^ in order to correct for multiple hypothesis testing, indicated that a total of 59 genes were down-regulated. Of these, 22 genes (37%) were identified as bound by the Hap2/3/5 complex by both CPD fingerprinting and ChIP-exo methods (Fig. 4F), a statistically significant overlap (*P* < 1 × 10^−34^). An additional 12 genes (20%) were identified as bound by CPD-seq only (Fig. 4F), again a significant overlap (*P* < 1 × 10^−18^). None of the remaining 25 down-regulated genes were identified as bound by ChIP-exo only (*P* > 0.05). Similar results were obtained when analyzing published expression data [50] for *hap3*Δ and *hap5*Δ mutant yeast (Supplementary Fig. S5). Taken together, these findings indicate that Hap2/3/5 binding sites identified by their CPD fingerprint are associated with many genes in functional categories regulated by Hap2/3/5 and that are down-regulated in *hap2*Δ, *hap3*Δ, or *hap5*Δ mutants. In contrast, target genes identified by ChIP-exo only did not show enrichment in these functional categories, and generally were not differentially expressed in *hap2*Δ, *hap3*Δ, or *hap5*Δ mutants.

### Hap2/Hap3/Hap5 binding peaks identified by ChIP-exo only are enriched in the coding regions of highly transcribed genes

Of the three ‘ChIP-exo only’ target genes linked to the ‘electron transport and membrane-associated energy conservation’ and deficiency in aerobic respiration functional categories (i.e. *ILV5, ACO1, PMA1*, see Fig. 4D), two of these (i.e. *ILV5, PMA1*) showed peaks of ChIP-exo reads throughout the coding region of the gene, not the promoter region (Supplementary Fig. S6A and B). Moreover, the Hap2/3/5 ChIP-exo peaks were spread widely throughout the *ILV5* and *PMA1* coding regions, instead of being tightly clustered, as observed for Hap2/3/5 binding sites in promoters (i.e. compare Supplementary Fig. S6A and B with Figs 2D and 3A). Notably, both of these genes are highly transcribed in yeast, with an estimated transcription frequency of 48.5 and 95 mRNA transcripts per hour for *ILV5* and *PMA1*, respectively [52]. In contrast, the median transcription frequency for all yeast genes is ∼2.0 mRNA transcripts per hour.

To test whether other “ChIP-exo only” target peaks occurred inside of genes, we analyzed the distribution of Hap2/3/5 binding sites identified by each method relative to the TSS of the nearest gene. This analysis indicated that Hap2/3/5 binding sites identified only by their CPD fingerprint were enriched in upstream promoter regions immediately adjacent to the TSS (i.e. from + 100 to −200 bp from the TSS; Fig. 4G, top panel). A similar enrichment in upstream promoter regions was observed for Hap2/3/5 binding sites identified by both CPD fingerprinting and ChIP-exo (Fig. 4G, middle panel), although these binding sites tended to be more distal to the TSS than the binding sites only identified by their CPD fingerprint. In contrast, Hap2/3/5 ChIP-exo only peaks were primarily enriched inside of genes, frequently at positions >700 bp from the TSS (Fig. 4G, bottom panel).

Previous studies have suggested that ChIP-seq experiments can be plagued by artifactual peaks in the coding regions of highly transcribed genes [12, 13]. Consistently, we observed many ChIP-exo only peaks associated with the coding regions of a number of highly transcribed genes, including *ADH1* (Supplementary Fig. S6C). Analysis of ChIP-exo only target genes revealed that these genes had significantly higher expression levels than the rest of the yeast genes (Supplementary Fig. S6D), particularly for target genes in which the ChIP-exo peak occurred in the coding region of the gene (median of 90 mRNA transcripts per hour, see Supplementary Fig. S6E). In summary, these results indicate that target genes identified by ChIP-exo only are not enriched for Hap2/3/5 regulated genes or functional categories, but are instead enriched in the coding regions of highly expressed genes.

### Mapping binding sites of other yeast ssTFs by CPD fingerprinting

We wondered whether other classes of yeast ssTFs also induce distinctive UV damage patterns at their binding sites that could be used for CPD fingerprinting. To address this question, we analyzed patterns of CPD induction in our cellular CPD-seq data relative to the naked DNA controls within the binding sites of 78 different yeast ssTFs, which were chosen because they have binding sites identified from published ChIP-exo data [36]. We then analyzed CPD induction patterns associated with each of the 78 different classes of TFBS using the UMAP algorithm [53].

**Figure 5.**
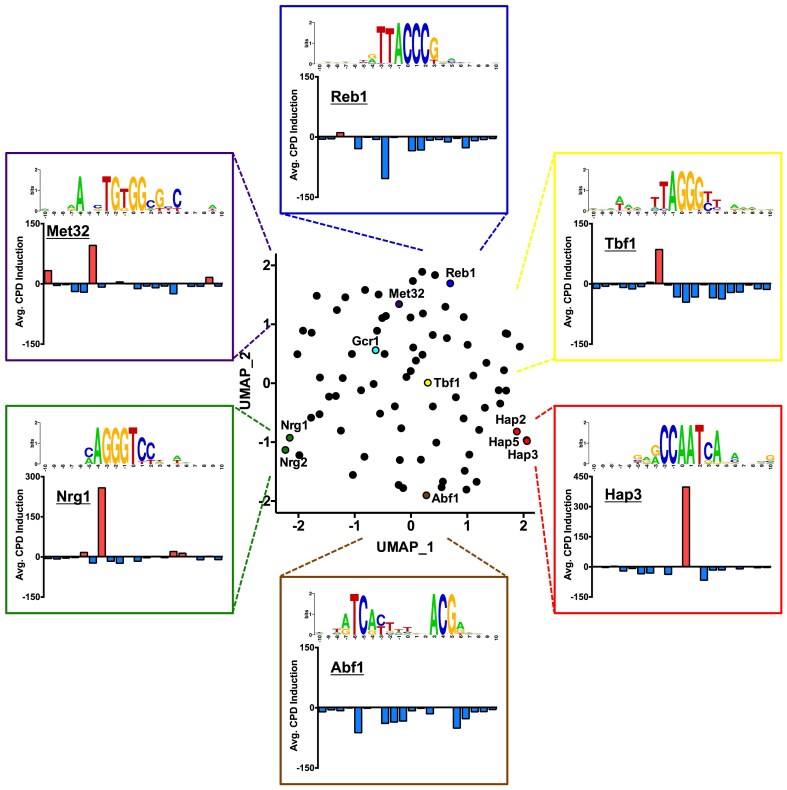
UMAP analysis of CPD induction patterns associated with binding sites for 78 different yeast ssTFs. Average CPD induction values in UV-irradiated WT cells relative to normalized naked DNA controls were analyzed between positions −5.5 to +5.5 relative to the center of each TFBS. The indicated ssTFs are highlighted and labeled on the UMAP graph, and the average CPD induction patterns of their binding sites are plotted. Sequence logos for each set of binding sites analyzed were generated using weblogo software [63].

The resulting UMAP plot indicated that the binding of yeast ssTFs to DNA induces a diverse array of UV damage patterns (Fig. 5). For example, some transcription factors showed peaks of CPD induction at specific locations in their binding sites (e.g. Nrg1, Tbf1, and Met32), while others (e.g. Reb1 and Abf1) primarily suppressed CPD formation in their binding sites (Fig. 5). In general, binding sites from related transcription factors clustered together (e.g. Nrg1 and Nrg2), indicating that these related proteins cause similar patterns of UV damage when bound to DNA. Many ssTFs in yeast are members of the C6 zinc cluster family of transcription factors, which bind pairs of CGG sequences with different spacing and/or orientation [54]. One of these ssTFs is Pdr1, which regulates genes involved in multidrug resistance [55]. Analysis of CPD-seq data at 19 known Pdr1 binding sites revealed a distinct pattern of CPD induction (Supplementary Fig. S7), suggesting that CPD fingerprinting may also be effective at mapping TFBS for this family of ssTFs.

Since CPDs only form at dipyrimidines (i.e. TT, TC, CT, and CC), a second consideration is to what extent CPD-forming dipyrimidine sequences are present in motifs bound by different classes of ssTFs. For example, 100% of the known Hap3 binding sites had dipyrimidines at two positions in the binding motif (i.e. CC and AA/TT in the CCAAT motif), and two other positions had dipyrimidines in least 75% of the known binding sites (Supplementary Fig. S8A and B). Similar analysis of binding sites for 77 other ssTFs indicated that on average ∼2.5 positions were dipyrimidines in 100% of the binding sites and ∼4.2 positions were dipyrimidines in at least 75% of the binding sites (Supplementary Fig. S8A and B). This analysis indicates that most ssTFs bound DNA motifs amenable for CPD fingerprinting.

**Figure 6.**
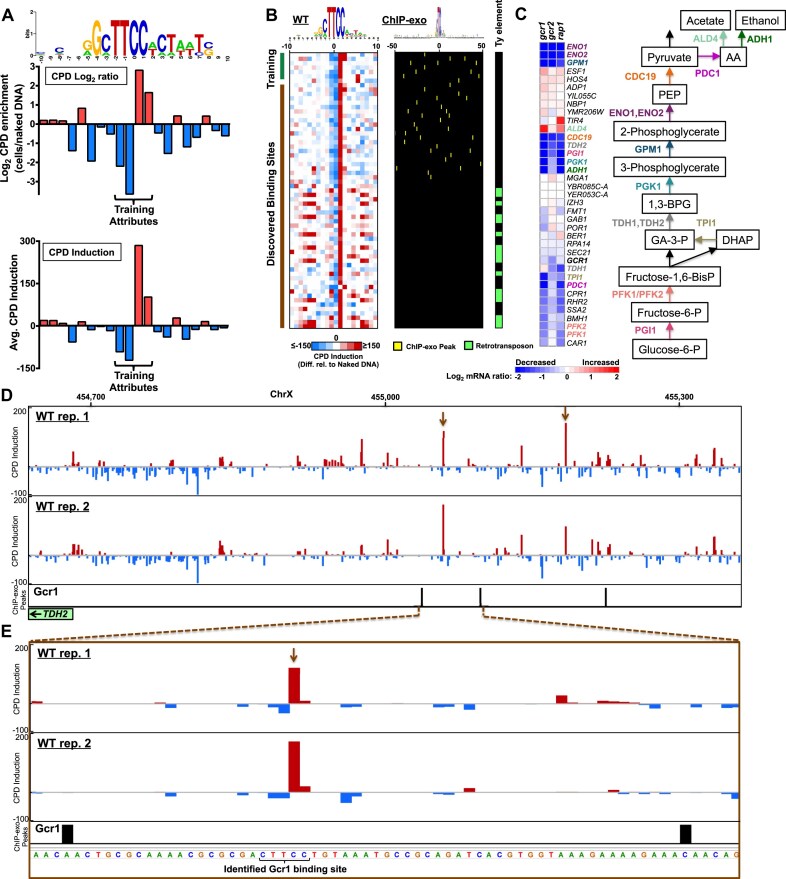
Identifying Gcr1 binding sites by learning their distinct CPD fingerprint. (**A**) Plot of log_2_ CPD enrichment (top graph) and average CPD induction (bottom graph) in UV-irradiated cells relative to the scaled naked DNA control for six known Gcr1 binding sites (based on published ChIP-exo data from [36]). Sequence logo in top panel was generated using the weblogo software [63]. (**B**) CPD induction values adjacent to 63 Gcr1 binding sites identified by the NN classifier. Each row depicts the CPD induction values of a single binding site. Rows are organized with the 6 known binding sites in the training data on top (green rectangle), 57 discovered binding sites not present in the training data (brown rectangle) at the bottom. Columns indicate the positions of damage induction relative to the center of the bound motif. Colors indicate the sign and magnitude of the damage induction, with red indicating higher CPD induction in cells relative to naked DNA, and blue indicating lower CPD induction in cells relative to the naked DNA control (see color bar). Top of the panel gives the sequence logo for the 63 identified binding sites, generated using the weblogo software [63]. Left panel indicates CPD induction data for WT cells, middle panel indicates the positions of ChIP-exo peaks within 50 bp of the 63 identified binding sites, derived from published ChIP-exo data for Gcr1 [36], and right panel indicates whether the binding site occurs in Ty element retrotransposon or LTR. (**C**) Left panel depicts changes in mRNA levels in *gcr1, gcr2*, and *rap1* mutant cells for genes associated with a Gcr1 binding site identified by CPD fingerprinting. All of these genes are associated with discovered Gcr1 binding sites, with the exception of TPI1, which is associated with only a known Gcr1 binding site. Blue color indicates a decrease in mRNA expression in the indicated mutants, while red indicates an increase in mRNA levels (see key at bottom of panel). Gene names are color-coded to indicate the step in the glycolysis pathway (see right panel) in which the enzyme they encode functions. mRNA expression data is from [65] and analyzed using RegulatorDB [66]. (**D**) CPD induction data for two UV-irradiated wild-type (WT) cell replicates relative to scaled naked DNA controls, as well as locations of called Gcr1 ChIP-exo peaks (bottom panel, data from [36]), in the promoter of the *TDH2* gene. Red bars indicate positive CPD induction in cells relative to naked DNA, and blue indicates negative CPD induction. Brown arrows indicate identified Gcr1 binding sites by CPD fingerprinting. Image generated using IGV [63]. (**E**) Same as panel *D*, except close-up of one of the identified Gcr1 binding sites, which is indicated by brown arrow.

While the Hap2/3/5 ssTF complex had 35 known TFBS based on published ChIP-exo data [36], many ssTFs have fewer mapped binding sites. For example, Gcr1 (see Fig. 5) is a key regulator of genes involved in glycolysis [7], but had only six known binding sites from ChIP-exo data available for training. Analysis of our CPD-seq data at these six Gcr1 binding sites indicated that CPD formation is induced primarily at positions +0.5 (CC dinucleotide) and +1.5, and suppressed at positions −1.5 (TT dinucleotide) and −0.5 (TC; see Fig. 6A). We trained a RF classifier with CPD induction and log ratio data derived from these six known Gcr1 binding sites (Fig. 6A) and 7000 negative examples, consisting of [C/A]TTCC sequences inside of yeast ORFs. The results indicated that the RF classifier had good accuracy overall (AUC of 0.9999), but misclassified 2 out of the 6 positive training instances when performing two-fold cross validation (Supplementary Fig. S9A). We tried three other commonly used machine learning algorithms (i.e. multilayer perceptron/NN, logistic regression, and Naïve Bayes). While Naïve Bayes performed worse on the training data (AUC ∼ 0.812, see Supplementary Fig. S9B), logistic regression misclassified only 1 out of the 6 positive training instances, and the NN misclassified none of the training instances (AUC of 1.0; Supplementary Fig. S9C). Similar analysis of the original Hap2/3/5 training and test data indicated that the NN classifier identified 73 out of the 75 binding sites predicted by RF (Supplementary Fig. S10).

We trained the NN classifier on the full Gcr1 training set and then used it to identify Gcr1 binding sites based on their CPD fingerprint. The NN classifier analyzed UV damage signatures at 56 312 [C/A]TTCC motifs across the yeast genome and identified a total of 63 Gcr1 binding sites. These 63 TFBS included all six of the known Gcr1 binding sites included in the training data, and 57 discovered binding sites (Fig. 6B). Of the 57 discovered TFBS, 23 binding sites had a Gcr1 ChIP-exo peak located within 50 bp, while many of the other 34 TFBS occurred in yeast Ty element retrotransposons (Fig. 6B). This is consistent with previous reports indicating that Gcr1 binding sites occur in the Ty retrotransposon long-terminal repeat (LTR) and regulate its expression [7, 56, 57]. Analysis of genes whose TSS is located within 700 bp of a discovered Gcr1 binding site revealed significant enrichment of genes annotated in the glycolysis and gluconeogenesis functional category (*P* < 1 × 10^−14^). These genes encoded enzymes involved in nearly every step of glycolysis (Fig. 6C). For example, the NN classifier identified two Gcr1 TFBS in the promoter of the *TDH2* gene (Fig. 6D and E), which encodes the key glycolytic enzyme glyceraldehyde-3-phosphate dehydrogenase. These Gcr1 TFBS were located within 50 bp of nearby Gcr1 ChIP-exo peaks (Fig. 6D and E); however, there were additional Gcr1 ChIP-exo peaks located much further away inside the coding region of the *TDH2* gene, which were not associated with a Gcr1 binding site identified by CPD fingerprinting (Supplementary Fig. S11). Since *TDH2* is a very highly expressed gene (98.9 mRNA transcripts per hour [52]), it is possible that these may reflect ChIP artifacts (see above). The discovered Gcr1 TFBS also showed significant enrichment of genes down-regulated in *gcr1* mutant cells (*P* = 9.6 × 10^−9^), including *TDH2* (Fig. 6C). The Gcr1 target genes identified by CPD fingerprinting were also in many cases down-regulated in *gcr2* and *rap1* mutant cells (Fig. 6C), consistent with previous reports suggesting that the Gcr1, Gcr2, and Rap1 ssTFs function to coordinately regulate many genes [58, 59]. Taken together, these findings indicate that CPD fingerprinting can be used to identify binding sites for multiple ssTFs (e.g. Hap2/3/5 and Gcr1) from the same CPD-seq dataset.

### Using CPD-capture-seq to map NF-Y binding sites in human promoter regions

The human homolog of the Hap2/3/5 complex is the NF-Y complex, consisting of the NF-YA, NF-YB, and NF-YC subunits. Like Hap2/3/5, NF-Y binds CCAAT motifs and regulates the transcription of many classes of target genes [6]. To characterize whether NF-Y binding to DNA induces a similar UV damage pattern as Hap2/3/5, we analyzed our published CPD-seq data derived from UV-irradiated normal human skin fibroblasts (NHF1) cells [21, 22]. We determined the average CPD induction in UV-irradiated NHF1 cells relative to the naked DNA control at 1659 NF-Y binding sites identified by ChIP-seq data from ENCODE that were associated with a DNase I hypersensitivity site (DHS) in skin melanocytes [38–40], as previously described [22]. The resulting analysis indicated that average CPD induction is elevated at NF-Y binding sites (Supplementary Fig. S12A), specifically at the TT dinucleotide opposite the AA in the CCAAT binding motif (Supplementary Fig. S12B). While this pattern matches the CPD fingerprint of Hap2/3/5 binding sites in yeast, there was a lower overall density of CPDs at these sites (Supplementary Fig. S12), due to the much larger size of the human genome, a potential barrier to using CPD-seq data to identify individual NF-Y binding sites.

**Figure 7.**
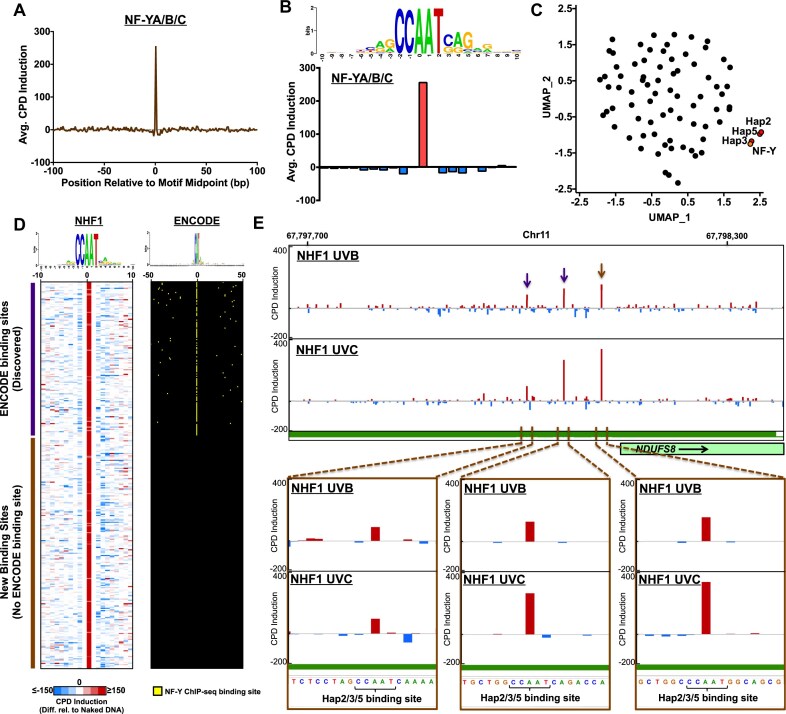
Using CPD-capture-seq and Random Forest to identify NF-Y binding sites in human cells. (**A**) Graph showing average CPD induction in normalized UVC- and UVB-irradiated NHF1 cells relative to UVC- and UVB-irradiated naked DNA controls associated with 156 NF-Y binding sites identified by ENCODE [38, 39] associated with a DNase I hypersensitivity (DHS) region in melanocytes [40] that overlapped with the central 360 bp of one (or more) of the capture regions. CPD-capture-seq data is from [41]. (**B**) Close up of average CPD induction data shown in panel A. Sequence logo was generated using weblogo software [63]. (**C**) UMAP analysis of CPD induction patterns associated with binding sites for 78 different yeast transcription factors (see Fig. 4), and human NF-Y binding sites derived from analysis of human CPD-capture-seq data. Average CPD induction data for positions −5.5 to +5.5 relative to the center of each transcription factor binding sites was analyzed using UMAP. The positions of data points corresponding to yeast Hap2/Hap3/Hap5 binding sites are indicated in red, and the NF-Y data point is indicated in orange. (**D**) Plot showing average CPD induction in normalized UVC- and UVB-irradiated NHF1 cells relative to UVC- and UVB-irradiated naked DNA controls for 337 NF-Y binding sites identified by the Random Forest algorithm. Each row corresponds to an individual binding site, and the colors indicate the magnitude of CPD induction (red) or CPD suppression (blue) in the UV-irradiated cells relative to the naked DNA control. Binding sites are sorted based on whether the identified binding site is located within 50 bp of an ENCODE NF-Y binding site identified by ChIP-seq analysis (i.e. ‘ENCODE binding sites (Discovered)’, top of panel) or not (i.e. ‘New binding sites (No ENCODE binding site)’, bottom of panel). The right panel indicates the location of the identified NF-Y ENCODE binding site relative to the identified NF-Y binding site identified by CPD-capture-seq. ENCODE data from [38, 39]. (**E**) Snapshot of CPD induction values in normalized UVB- or UVC-irradiated NHF1 cells relative to UVB- or UVC-irradiated naked DNA controls, associated with three identified NF-Y binding sites identified by Random Forest analysis of human CPD-capture-seq data. Dark green rectangles represent the location of capture region, while the light green rectangle with black outline indicates location of the *NDUFS8* gene. Arrows indicate locations of identified NF-Y binding sites, with purple arrows indicating NF-Y binding sites previously identified by ENCODE, and the brown arrow indicating a new binding site identified only by CPD fingerprinting. Bottom panels depict close-ups of CPD induction at each identified NF-Y binding site. Images generated using IGV [35].

To overcome this challenge, we leveraged our published CPD-capture-seq data [41]. CPD-capture-seq enriches for CPD-seq reads in a set of ∼4000 capture regions, comprising ∼2.8 million base pairs of the human genome primarily associated with promoters and other regulatory elements [41]. We identified 156 NF-Y ENCODE binding sites associated with DHS regions that overlapped with the central 360 bp of one (or more) of these capture regions. Analysis of normalized CPD-capture-seq data from UVB- or UVC-irradiated NHF1 cells relative to UVB- or UVC-irradiated naked DNA controls revealed a similar pattern of average CPD induction associated with NF-Y binding sites in capture regions (Fig. 7A,B). Again, CPDs levels were specifically induced at the central AA/TT base pairs in the bound CCAAT motif (Fig. 7B). Importantly, the overall CPD density was much higher in the CPD-capture-seq data, being roughly equivalent to the densities observed in UV-irradiated yeast cells.

We used UMAP to compare damage patterns at NF-Y binding sites in human cells to damage patterns associated with yeast TFBS. This analysis indicated that the NF-Y CPD fingerprint closely resembled those of yeast Hap2/Hap3/Hap5 binding sites (Fig. 7C). Based on this analysis, we hypothesized that the RF model developed and validated for the yeast Hap2/3/5 complex could be used to identify NF-Y binding sites in captured regions of the human genome using our CPD-capture-seq data.

To test this hypothesis, we identified 1859 CCAAT motifs located in the central 360 bp of a capture region, and used the Random Forest algorithm to identify bound motifs using the CPD-capture-seq data. This analysis identified 337 of the CCAAT motifs as predicted NF-Y binding sites, each of which had the expected damage pattern associated with NF-Y binding (Fig. 7D). Of these, 134 identified binding sites were within 50 bp of an NF-Y bound motif (Fig. 7D) based on ENCODE ChIP-seq data. Three of the identified NF-Y binding sites occurred in the promoter of the NADH ubiquinone oxidoreductase core subunit S8 (*NDUFS8*) gene, which is involved in mitochondrial electron transport and NADH reduction (Fig. 7E). All three identified binding sites show clear CPD induction in UVB- and UVC-irradiated NHF1 cells relative to the matching naked DNA controls (Fig. 7E). The two more distal NF-Y binding sites (indicated by brown arrows in Fig. 7E) were identified by previous ENCODE ChIP-seq data. In contrast, the identified binding site closest to the *NDUFS8* TSS (indicated by purple arrow in Fig. 7E) had not been previously identified in analysis of ENCODE ChIP-seq data, even though this binding site showed the strongest UV damage induction. Taken together, these findings suggest that analysis of damage fingerprints from CPD-capture-seq data can be used to identify human NF-Y binding sites.

## Discussion

Deciphering transcriptional networks requires accurate methods to identify TFBS in cells. Here, we show that machine learning algorithms can recognize characteristic UV damage fingerprints associated with DNA-binding by ssTF proteins, and these damage fingerprints can be used to map TFBS across the genome of yeast and human cells. Application of this methodology to the yeast Hap2/3/5 complex identified ∼25 new binding sites missed by previous genome-wide ChIP experiments [36, 48]. Moreover, we show that the same experimental approach can be used to identify NF-Y binding sites in promoters and enhancer regions in human skin cells by exploiting the high sequencing depth associated with human CPD-capture-seq data [41]. Taken together, these results indicate that UV damage mapping, coupled with machine learning, could be a powerful approach to decipher transcriptional networks in eukaryotic cells.

Multiple lines of evidence support the conclusion that the Hap2/3/5 binding sites identified by CPD-seq reflect bona fide Hap2/Hap3/Hap5 binding events in yeast cells. First, these binding sites showed a clear and consistent damage pattern in UV-irradiated cells, but not UV-irradiated naked DNA controls. Second, analysis of this UV damage signature using the Random Forest algorithm in cross-validation experiments faithfully identified known Hap2/3/5 binding sites, but not control CCAAT motifs. Third, this UV damage pattern was eliminated at all identified binding sites in *hap5*Δ mutant cells, in which the Hap2/3/5 complex is unable to bind DNA, even though the *hap5*Δ mutant data was not used in training or predicting binding sites using the RF model. Finally, the identified target genes, including those identified by a CPD fingerprint only and not ChIP-based methods, are enriched for known Hap2/3/5 regulated genes and functional categories, and their mRNA levels tend to be differentially expressed in yeast mutants lacking a functional Hap2/3/5 complex.

In contrast, the Hap2/3/5 target genes identified by ChIP-based methods, but not associated with a characteristic CPD fingerprint, are not significantly enriched for known functional categories or differentially expressed in Hap2/3/5 mutants, but instead are enriched in the coding regions of highly transcribed genes. These findings are consistent with previous reports suggesting that ChIP-based methods tend to yield artifactual signals at highly expressed genes [12, 13]. Previous studies indicate that formaldehyde crosslinking is enhanced in transient single-stranded DNA regions [60], which are often associated with ongoing transcription or transcription-associated R-loops. One possibility is that non-specific DNA interactions associated with the facilitated diffusion process, a mechanism by which DNA-binding proteins search for sequence-specific binding sites [61, 62], may be trapped by formaldehyde cross-linking when these binding intermediates occur in highly transcribed genes. In contrast, the CPD fingerprint method described here would not detect such binding intermediates, since it measures alterations in UV damage formation arising from DNA conformational changes in a natively bound TFBS.

A key question is why many of the Hap2/3/5 binding sites identified by our CPD-seq data were missed by previous studies using ChIP-based methods [36, 48], even though the same (or similar) yeast strain background (BY4741) and growth conditions (YPD media) were used. Since formaldehyde crosslinking efficiency is dependent upon DNA sequence [60], one possibility is that differences in flanking DNA sequence context could potentially explain this discrepancy. However, preliminary analysis did not reveal any significant sequence differences between the CPD fingerprint only binding sites and the binding sites identified by both ChIP-exo and CPD fingerprinting (Supplementary Fig. S13). Our analysis indicates that binding sites identified by CPD fingerprinting only are enriched immediately adjacent to the TSS of genes (Fig. 4G). Since RNA polymerase II and other components of the large pre-initiation complex (PIC) are enriched at the TSS [36], it is possible that epitope masking and/or crosslinking interference could potentially explain these results.

Our analysis also indicates that different classes of ssTFs induce distinct patterns of UV damage at their DNA binding sites and most have sufficient numbers of lesion-forming dipyrimidine sequences to learn ssTF-specific CPD fingerprints. A potential limitation is that our findings suggest that the RF algorithm may be less effective at learning CPD fingerprints of ssTFs with fewer known binding sites that are available to be used as training examples. However, we show that in such cases the multilayer perceptron/NN algorithm may be an excellent alternative approach. In the case of Gcr1, which had only six known binding sites available for training, the NN classifier identified 57 additional Gcr1 binding sites associated with expected categories of genes (i.e. genes that function in glycolysis and Ty retrotransposons), many of which were also down-regulated in *gcr1* mutant cells. For ssTFs with a larger number of positive training examples (e.g. Hap2/3/5), RF and NN identified similar sets of binding sites, although NN identified 17 additional Hap2/3/5 binding sites, likely due to its increased sensitivity to detect binding sites associated with weaker CPD fingerprints. Preliminary analysis indicates that these 17 additional Hap2/3/5 binding sites are associated with four genes annotated in the deficiency in aerobic respiration functional category (i.e. *TIM11, ACO1, COX7*, and *CIT1*), and two of these (*TIM11* and *CIT1)* are down-regulated in *hap2*Δ mutant cells. It is possible that more complex machine learning methods may do even better in such cases, particularly for ssTFs with more subtle CPD fingerprints. These findings suggest the intriguing possibility that with appropriate computational models of ssTF CPD fingerprints, a single UV damage mapping experiment could simultaneously elucidate the genome-wide binding profiles of many (or all) ssTFs.

ChIP methods exploit protein-DNA crosslinks induced by formaldehyde to map protein binding sites across the genome. Here, we show that mapping a different form of DNA damage induced by UV light can be used in conjunction with machine learning methods to identify protein-DNA binding events at single-nucleotide resolution in living cells. Since not only cells but intact organisms can be exposed to UV light, and the necessary UV dose can typically be delivered in tens of seconds, we envision that the method described herein could be used in future experiments to characterize ssTF binding dynamics in a variety of cellular, organismal, and environmental contexts.

## Supplementary Material

gkaf1014_Supplemental_File

## Data Availability

The yeast CPD-seq data described in this manuscript has been deposited in the Gene Expression Omnibus (GEO) database (https://ncbi.nlm.nih.gov/geo/) under accession number GSE292650. Custom software code used for this analysis can be found through Zenodo at https://doi.org/10.5281/zenodo.16921788.
