# Supplementary material for: Mapping transcription factor binding sites by learning UV damage fingerprints
Source: Nucleic Acids Res. 2025 Oct 14;53(19):gkaf1014. doi: 10.1093/nar/gkaf1014 (PMC12526043; doi:10.1093/nar/gkaf1014)
Supplement: gkaf1014_Supplemental_File [file gkaf1014_supplemental_file.pdf]

Supplementary Materials and Figures

Supplementary Materials and Methods

Yeast strains

The *hap5Δ* strain was generated via homologous recombination-mediated replacement of *hap5* with the *TRP1* selectable marker in MP019 strain (BY4741 background), and the knockout was confirmed by PCR screening. BY4741 was used for WT CPD-seq experiments.

Yeast UV exposure

Yeast cells (i.e., BY4741 [WT] and *hap5*Δ) were grown to an OD_{600} ~0.8 in yeast peptone dextrose (YPD) media at 30°C, harvested by centrifugation, resuspended in sterile water, and exposed to 125J/m^2 UVC light based on our previous calibration. Cells were recollected and spun down for genomic DNA (gDNA) isolation via the phenol chloroform isoamyl alcohol (PCI) method and ethanol precipitated (see below). Isolated yeast genomic DNA (i.e., naked DNA control) was resuspended in water and aliquoted in 10uL spots on a microscope slide coverslip. Coverslips with DNA were placed on ice to avoid evaporation during UV exposure. Naked DNA was exposed to 90J/m^2 UVC light, according to our previous calibrations, but likely received a slightly higher dose because the coverslips were raised a few inches above the surface in an ice bucket. DNA was then recollected in a fresh sterile tube for subsequent CPD-seq library preparation.

Yeast genomic DNA isolation

Yeast cells harvested by centrifugation after UV exposure for genomic DNA (gDNA) isolation. gDNA was isolated via the phenol chloroform isoamyl alcohol (PCI, 25:24:1) method and ethanol precipitated, as previously described (1). Briefly, ~15mL of yeast cells were spun down at 3,000rpm for 5 minutes in sterile 50mL conicals and resuspended in 350uL yeast DNA lysis buffer (2% [vol/vol] Triton X-100, 1% SDS, 100mM NaCl, 10mM Tris-HCl, pH 8.0, and 1mM EDTA), 250uL PCI, and 250uL acid-washed beads. This solution was then vortexed at the highest setting for four minutes and 200uL of TE (10mM Tris-HCl, pH 7.5, and 1mM EDTA) was added. Cell lysates were then inverted to mix several times and centrifuged at 13,000 rpm for ten minutes to separate the DNA-containing aqueous layer. DNA was precipitated out of supernatant solution by addition of 1mL 100% ethanol and incubated at -20˚C for at least 15 minutes. DNA was recovered via centrifugation and washed with 80% ethanol. Pelleted DNA was resuspended in 200uL TE buffer and incubated with 2uL RNase A at 37˚C for 1 hour to degrade contaminating RNA. A second PCI and ethanol extraction was performed to further purify the DNA. Resultant DNA was resuspended in 100uL sterile molecular biology grade water.

Oligonucleotide sequences

The following oligonucleotides were used as PCR primers for the CPD-seq experiments:

>RAPID0 (i5)
AATGATACGGCGACCACCGAGATCTACACAGGCTATAACACTCTTTCCCTACACGA
CGCTCTTCCGATCT
>RAPID2 (i7)
CAAGCAGAAGACGGCATACGAGATGAGCCAATGTGACTGGAGTTCAGACGTGTGC
TCTTCCGATC
>RAPID3 (i7)
CAAGCAGAAGACGGCATACGAGATCAGATCTGGTGACTGGAGTTCAGACGTGTGC
TCTTCCGATC
>RAPID4 (i7)
CAAGCAGAAGACGGCATACGAGATTGTGAAGAGTGACTGGAGTTCAGACGTGTGC
TCTTCCGATC
>RAPID5 (i7)
CAAGCAGAAGACGGCATACGAGATACAGTGGTGTGACTGGAGTTCAGACGTGTGC
TCTTCCGATC

Validation of Hap2/3/5 target genes
To identify Hap2/3/5 target genes, each Hap2/3/5 binding site was assigned to the closest TSS of a neighboring gene located within 700 bp of the binding site midpoint, using TSS coordinates from (2). Gene ontology and functional categorization analysis of Hap2/3/5 target genes was performed FunSpec (3). Changes in mRNA levels in *hap2*Δ, *hap3*Δ, or *hap5*Δ mutants are from Kemmeren et al. (4), and were analyzed using data from the RegulatorDB database (5) using a threshold of $P < 1x10^{-6}$ and $\log_2$ ratio ≤ -0.5 (down-regulated) or $\log_2$ ratio ≥ 0.5 (up-regulated). Analysis of ChIP-chip data from (6) were analyzed using the Ceres database (7) with medium binding criteria ($P < 0.005$) and located up to 1000 bp upstream of the target gene, cut off at adjacent genes. ChIP-exo only Hap2/3/5 binding peaks were combined if they were within 100 bp of a neighboring Hap2/3/5 ChIP-exo peak.

Analysis of Gcr1 target genes
We identified Gcr1 target genes similar to the protocol described for Hap2/3/5 target genes above, except if more than one gene had their TSS within 700 bp of an individual Gcr1 binding site, all were labeled as Gcr1 targets. Analysis of gene functional categories was performed using FunSpec (3) as described above. Changes in gene expression of identified target genes in *gcr1*, *gcr2*, and *rap1* mutant cells were analyzed with RegulatorDB (5) with default fold-change and P-value thresholds using data from Hu et al. (8,9), since the Kemmeren et al. data set did not contain expression data for a *gcr1* mutant.

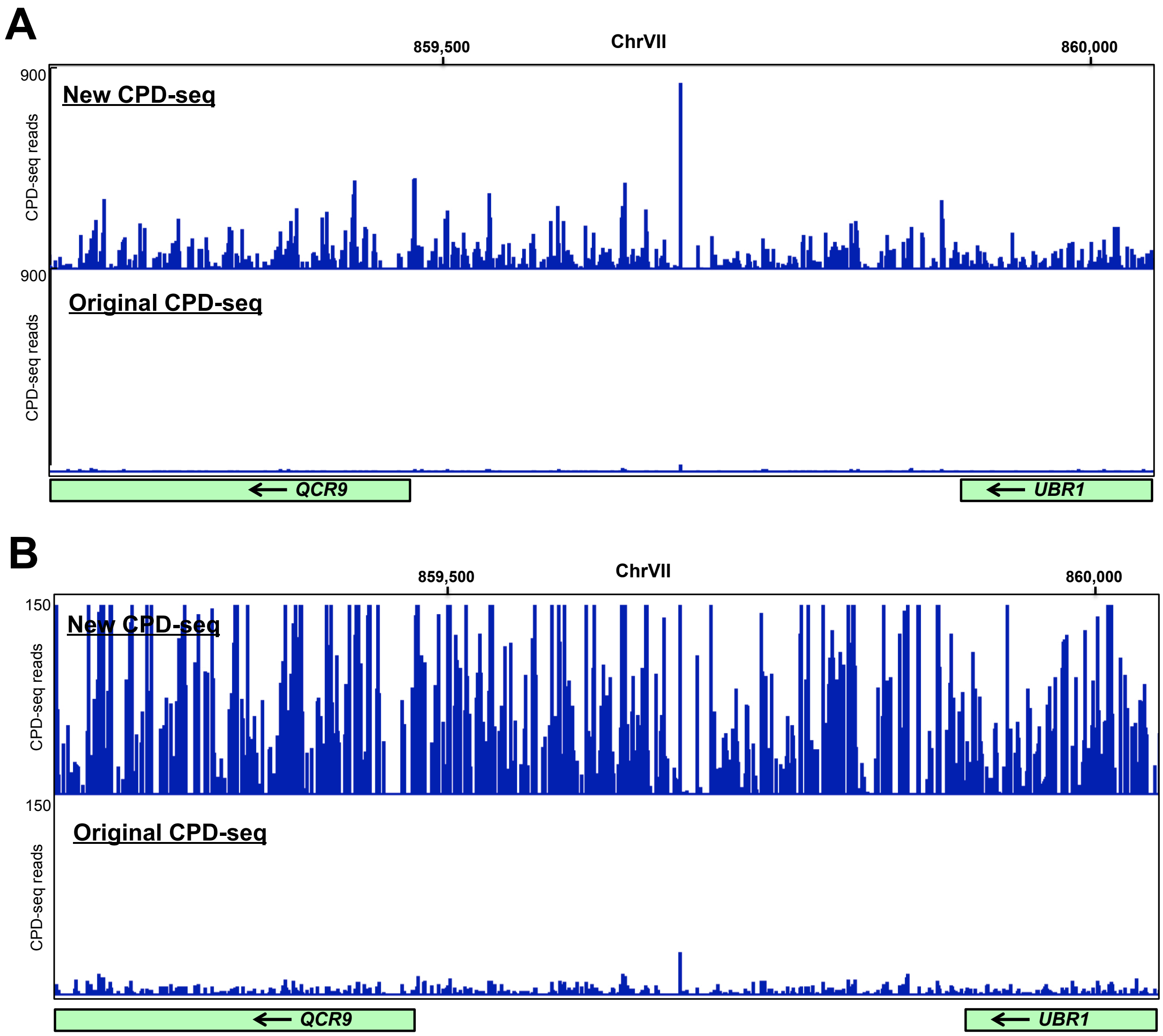

Supplementary Figure S1. (*A*) Comparison of CPD-seq read density from new deep CPD-seq sequencing data (top panel) relative to our previously published yeast CPD-seq data (bottom panel). Previous CPD-seq data from (10). (*B*) Same as panel A, except the Y-axis is zoomed in six-fold. Figure generated using the Integrative Genomics Viewer (IGV) (11).

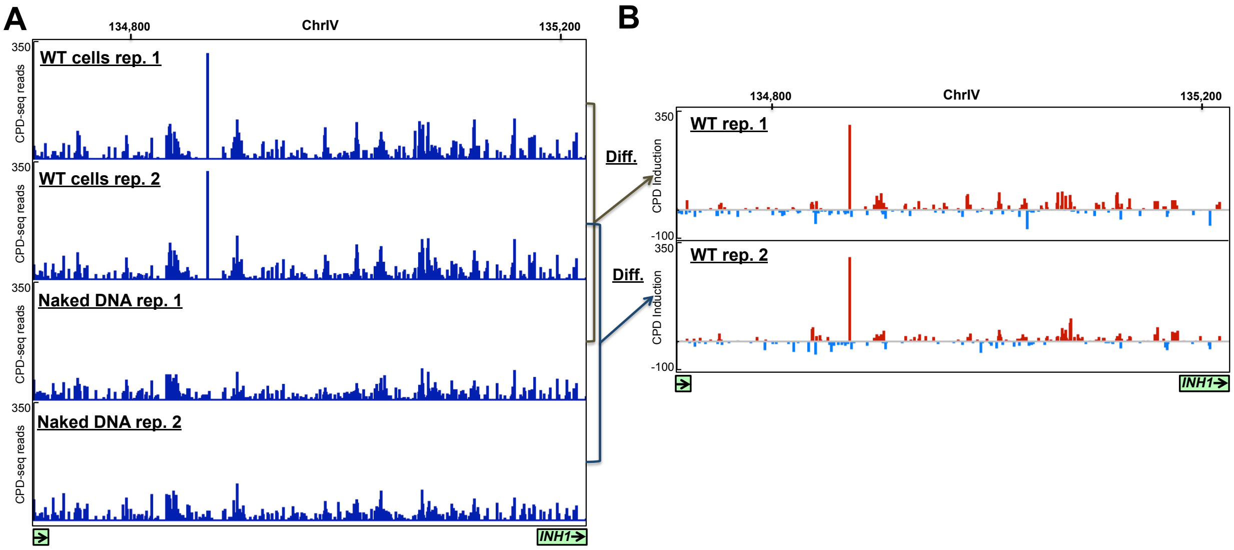

Supplementary Figure S2. (*A*) IGV snapshot of normalized counts of CPD-seq reads in two UV-irradiated wildtype (WT) yeast cell replicates (rep1 and rep2, top two panels) and corresponding UV-irradiated naked DNA replicates (two bottom panels) in the promoter region of the *INH1* gene (depicted with green rectangle at bottom right of figure). (*B*) Visualization of corresponding CPD induction values derived from subtracting the difference in the normalized WT cellular and naked DNA control CPD counts at each position. Figure generated using IGV (11).

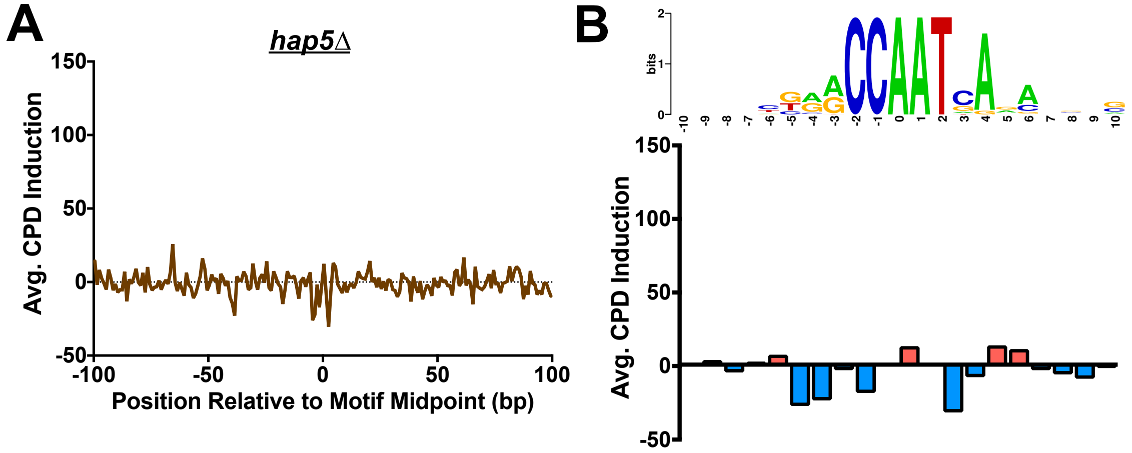

Supplementary Figure S3. Analysis of average CPD induction at Hap2/Hap3/Hap5 binding sites in UV-irradiated *hap5*Δ cells. (*A*) Graph of average CPD induction in *hap5*Δ mutant cells relative to a naked DNA control in DNA adjacent to 35 known Hap2/Hap3/Hap5 binding sites, based on published ChIP-exo data (12). Average CPD induction was calculated based on the difference in normalized CPD counts in *hap5*Δ mutant cells relative to a naked DNA control. (*B*) Close-up of average CPD induction in *hap5*Δ mutant cells relative to a naked DNA control at 35 known binding sites. Sequence logo was generated using weblogo software (13).

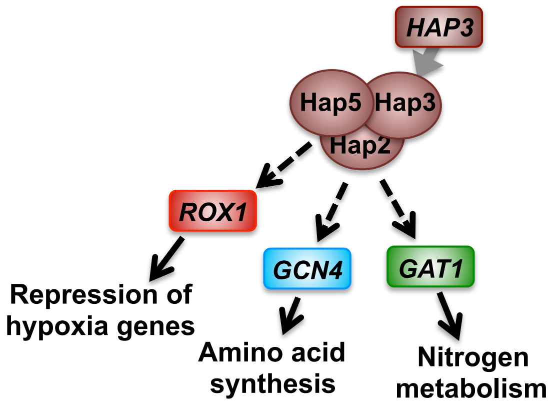

Supplementary Figure S4. Network of sequence-specific transcription factors whose promoter regions are bound by the Hap2/3/5 complex based on previous ChIP-exo data (12). Rectangles are genes/promoter regions and circles indicate encoded proteins. Black arrows indicate regulatory interactions; the dashed lines indicate that only two out of the three complex subunits show significant binding to each of the target genes (i.e., *ROX1, GCN4*, and *GAT1*). Gray arrow indicates that the *HAP3* gene encodes the Hap3 subunit.

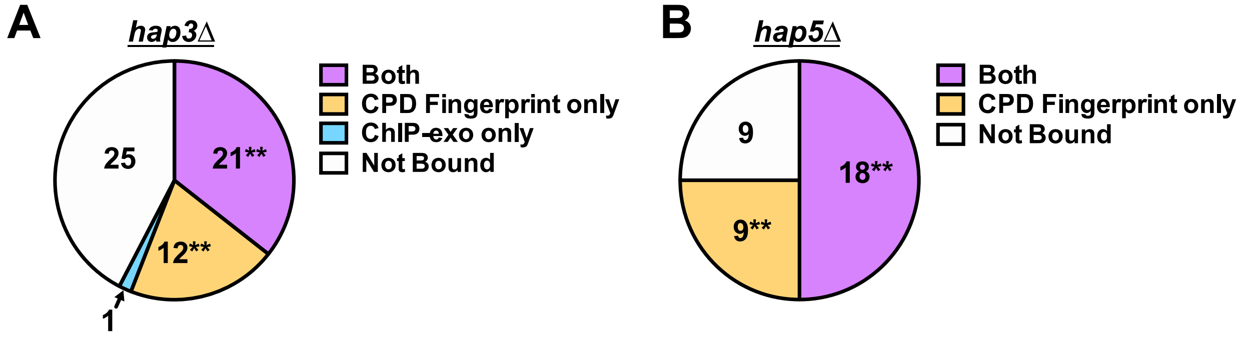

Supplementary Figure S5. (*A,B*) Pie chart indicating the number of down-regulated genes ($P < 1x10^{-6}$, log_2 ratio ≤ -0.5) in (*A*) *hap3*Δ and (*B*) *hap5*Δ mutant cells that contain binding sites identified by both CPD Fingerprinting and ChIP-exo ('Both'), CPD Fingerprint only, by ChIP-exo only, or by neither method ('Not Bound'). **Indicates a significant overlap of Hap2/3/5 target genes with the genes down regulated in mutant cells; P < 0.0001, based on the hypergeometric distribution.

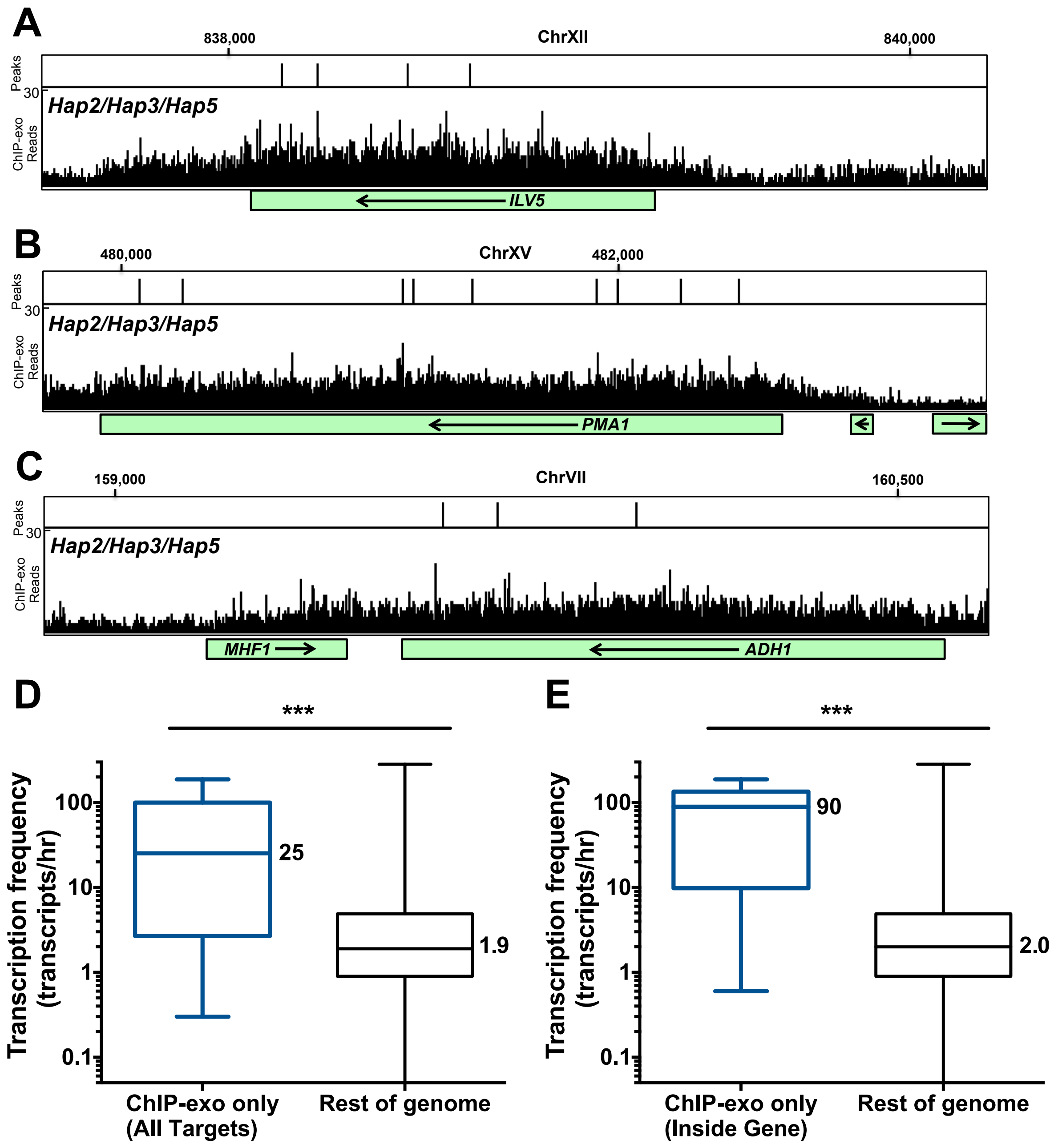

Supplementary Figure S6. (*A*-*C*) Plot of ChIP-exo only peaks occurring in the coding regions of (*A*) ILV5, (*B*) PMA1, and (*C*) ADH1 genes. ChIP-exo peaks for Hap2, Hap3, and Hap5 experiments and ChIP-exo reads from the same experiments are shown in aggregate. ChIP-exo data is from (12). (*D*) Box plot showing transcription frequency in transcripts per hour for genes associated with ChIP-exo only target peaks relative to the rest of the genome. The median transcription frequency values for each data set is indicated. Transcription frequency data is from (14). ***P < 0.0001 based on Mann-Whitney test. (*E*) Same as panel D, except just comparing genes with ChIP-exo only peaks that occur inside a gene.

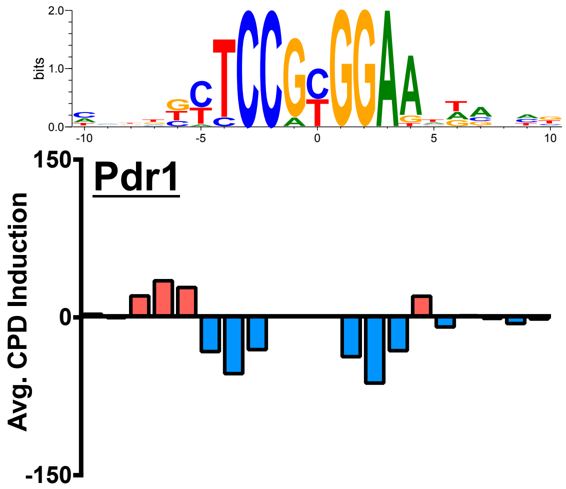

Supplementary Figure S7. Pattern of average CPD induction in UV-irradiated yeast cells relative to scaled UV-irradiated naked DNA control at 19 known Pdr1 binding sites, derived from published ChIP-exo data (12). Total CPD induction at each position was divided by the number of Pdr1 binding sites. The sequence logo shown in the top panel was generated using the weblogo software (13).

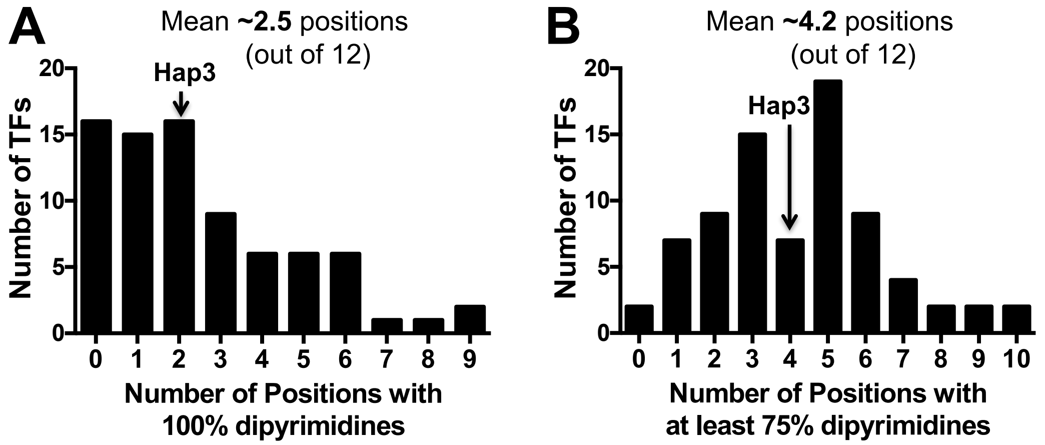

Supplementary Figure S8. Histograms showing the number of ssTFs in yeast (out of 78 total) with the indicated number of positions in their binding motif in which a dipyrimidine occurs in (*A*) 100% or (*B*) at least 75% of known binding sites. Binding site data for the 78 ssTFs is derived from published ChIP-exo data (12). The numbers for Hap3 binding sites are indicated.

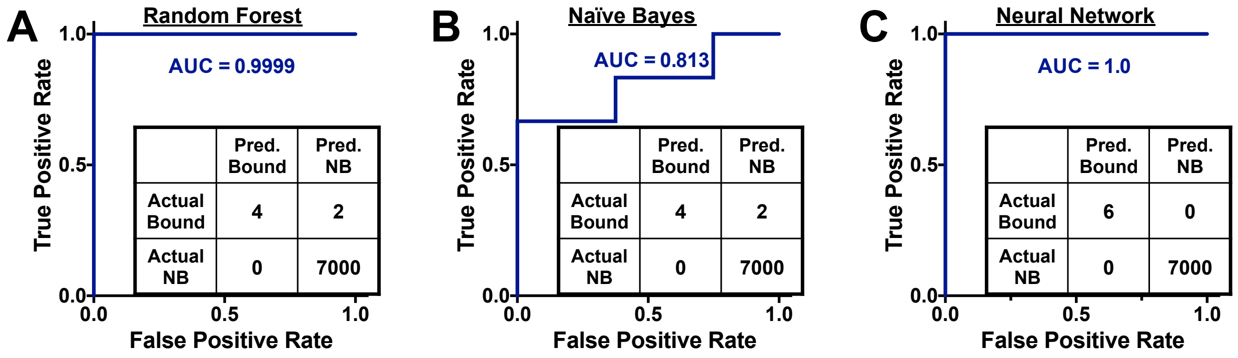

Supplementary Figure S9. (*A-C*) Graphs showing ROC curve and confusion matrix results for the indicated machine learning algorithm applied to a training set of CPD-seq data for six known Gcr1 TFBS and 7000 control [C/A]TTCC sequence motifs under two-fold cross-validation. NB = not bound, AUC = area under curve.

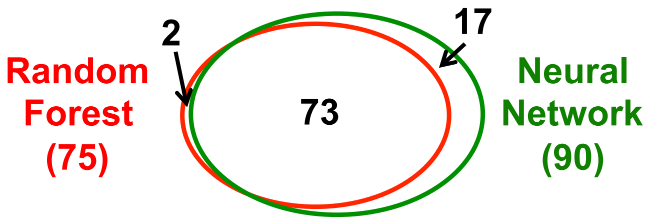

Supplementary Figure S10. Overlap in Hap2/3/5 binding sites identified by Random Forest and multilayer perceptron/Neural Network machine learning methods is depicted.

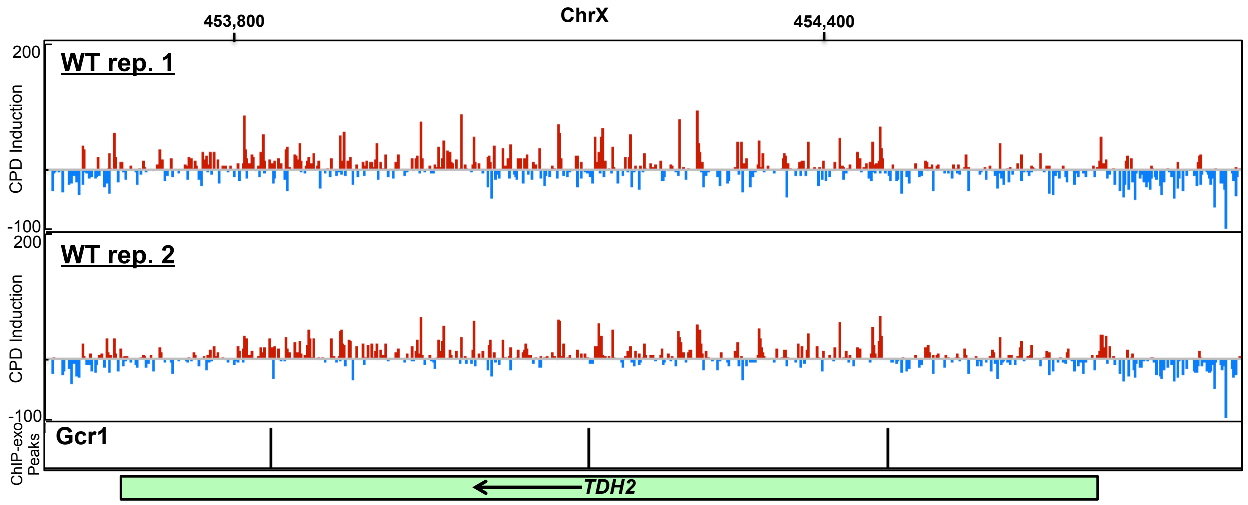

Supplementary Figure S11. Gcr1 ChIP-exo peaks that are dispersed in the highly expressed TDH2 gene are not associated with Gcr1 binding sites identified by CPDs fingerprinting. ChIP-exo peaks derived from (12). Image generated using IGV (11).

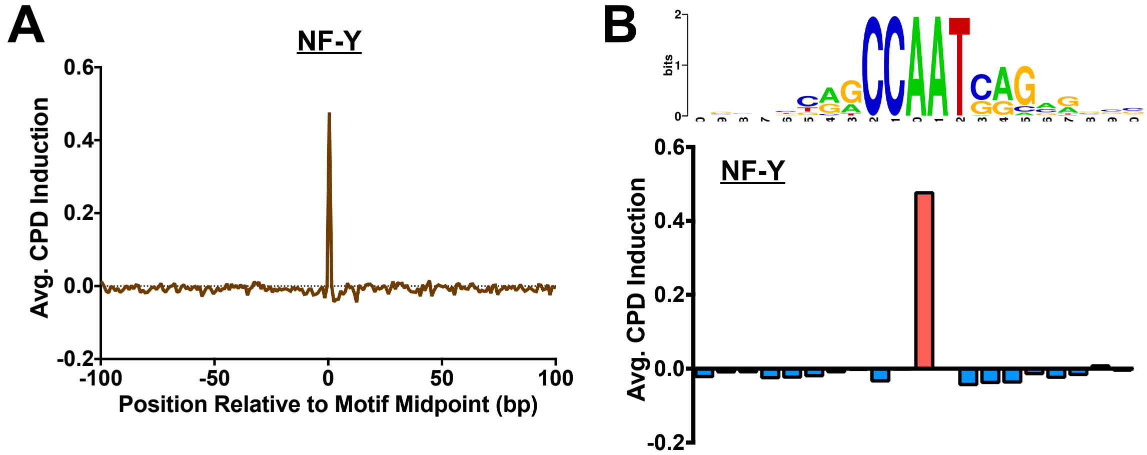

Supplementary Figure S12. Average CPD induction at NF-Y binding sites in UV-irradiated immortalized normal human fibroblasts (NHF1) cells. (*A*) Graph of average CPD induction in NHF1 cells relative to a naked DNA control in DNA adjacent to 1659 NF-YA/B binding sites, based on analysis of motifs in published ChIP-seq data from ENCODE (15,16), and located in a DNase I hypersensitivity (DHS) region in melanocytes (17). Average CPD induction was calculated based on the difference in normalized CPD counts in cells relative to a naked DNA control, using published CPD-seq data for NHF1 cells and naked DNA controls (18,19). (*B*) Close-up of average CPD induction in NHF1 cells relative to a naked DNA controls at 1659 NF-YA/B binding sites. Sequence logo was generated using weblogo software (13).

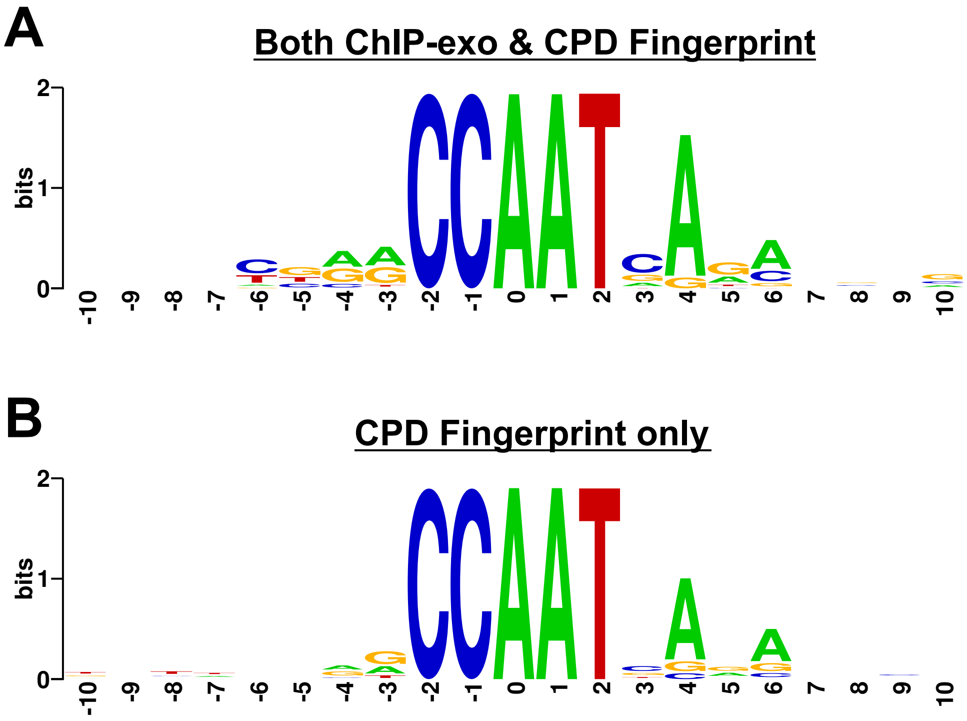

Supplementary Figure S13. (*A,B*) Comparison of DNA sequence logos of Hap2/Hap3/Hap5 binding sites identified by (*A*) both CPD Fingerprinting and ChIP-exo and by (*B*) CPD Fingerprint only. Sequence logos were generated using the weblogo software (13).

Supplementary Table S1: Definitions of key biological terms in manuscript.

Term	Definition
**Cyclobutane pyrimidine dimer (CPD)**	Major form of UV damage involving the physical linkage between adjacent pyrimidine bases in DNA.
**Sequence-specific transcription factors (ssTF)**	Regulatory proteins that bind a specific sequence of DNA bases.
**Transcription factor binding site (TFBS)**	Specific DNA sequences bound by sequence-specific transcription factor proteins, typically for the purpose of gene regulation.
**CPD-seq**	Cyclobutane pyrimidine dimer-sequencing; a technique used to map the formation of CPDs genome-wide at single nucleotide resolution.
**CPD-capture-seq**	Similar to CPD-seq, but only sequences specific regions of interest across a genome. Typically used in human cells or genomic DNA due to the large size of the human genome.
**DNase I hypersensitivity sites (DHS)**	Specific regions of the genome that are more accessible to protein binding and therefore more readily cleaved by DNA endonuclease DNaseI.
**Formaldehyde crosslinking**	The use of formaldehyde treatment to covalently affix proteins to DNA.
**Glycolysis**	Metabolic pathway that converts glucose into pyruvate to produce cellular energy in the form of adenosine triphosphate (ATP).
**Gluconeogenesis**	Metabolic pathway that generates glucose from non-carbohydrate substrates (e.g., lactate or glycerol).
**Epigenetic modifications**	Changes to DNA or DNA-associated proteins (e.g., histones) that alter gene expression without modifying the DNA sequence itself (i.e., cytosine methylation).
**Promoter**	DNA region immediately neighboring the gene that contains TFBS and other regulatory sequences that control gene expression.
**Enhancer regions**	More distant genomic regions containing TFBS that regulate gene expression.
**Open reading frame (ORF)**	Protein-coding segment of gene.

**Intragenic motifs**	DNA sequence motifs located inside a gene.
**Aerobic respiration**	Process by which energy is generated by converting end products of glycolysis in the presence of oxygen into carbon dioxide and water.
**Mitochondria**	Organelle in the cell that, among other functions, helps to generate energy through the process of aerobic respiration. The powerhouse of the cell.
**Mitochondrial electron transport chain**	A series of transmembrane proteins spanning the inner mitochondrial membrane that concertedly function to transport electrons across a gradient, ultimately leading to ATP (energy) production during the process of aerobic respiration.
**Mitochondrial cytochrome c oxidase**	Final enzyme in the mitochondrial electron transport chain.
**Ubiquinol cytochrome c reductase complex**	Critical protein in mitochondrial electron transport chain.
**Target genes**	Genes that are regulated by ssTFs.
**mRNA expression data**	messenger RNA (mRNA) data can measure the rate of transcription and show which genes are turned 'on' and 'off' during the process.
**Tricarboxylic acid (TCA) cycle**	A series of chemical reactions to extract energy from molecules. A process crucial to aerobic respiration.
**Transcriptional activators**	ssTFs that positively regulate transcription.
**Hypoxia**	A low oxygen environment.
**Hypoxia genes**	Genes that are expressed and/or function in a low oxygen environment.
**Coding region**	Region of a gene that codes for a specific protein.
**Retrotransposon**	A mobile genetic element usually present in multiple copies in a genome.
**Ty element retrotransposons**	A type of retrotransposon present in *S. cerevisiae.*
**Ty retrotransposon long-terminal repeat (LTR)**	A region of the retrotransposon that often functions as a promoter.
**Fibroblasts**	Most common type of cell found in connective tissue.
**Melanocytes**	A mature melanin-producing cell, especially in the skin.

**ENCODE**	The Encyclopedia of DNA Elements database. A public research project aimed at compiling a comprehensive list of functional cellular parts in the human genome. Contains data from various experiments such as ChIP-seq.
**NADH ubiquinone oxidoreductase core subunit S8 (NDUFS8)**	Protein that is a crucial component of mitochondrial complex I that functions as the first protein in the electron transport chain.
**NADH reduction**	The process by which NAD+ is reduced to NADH, involving the gain of electrons and a hydrogen ion to be processed into cellular energy in the mitochondrial electron transport chain during aerobic respiration.
**Transcription-associated R-loops**	Three-stranded nucleic acid structures that form while transcription is occurring in which the nascent RNA pairs to the actively transcribed strand of DNA and the non-transcribed strand of DNA is left single-stranded.
**BY4741**	Wild-type *Saccharomyces cerevisiae* (Baker's yeast) strain.
**YPD**	Yeast extract Peptone Dextrose media used to culture yeast strains.
**Pre-initiation complex (PIC)**	A complex of many proteins required to trigger the process of transcription initiation.
**Epitope masking**	The phenomenon in which an antibody is unable to bind its antigen target due to another protein or molecule blocking access to the antigen.
**Sequence logos**	A visual representation of a consensus DNA binding sequence where the height of a base at each position in the motif represents the information content.

Supplementary References

1. Bohm, K.A., Hodges, A.J., Czaja, W., Selvam, K., Smerdon, M.J., Mao, P. and Wyrick, J.J. (2021) Distinct roles for RSC and SWI/SNF chromatin remodelers in genomic excision repair. *Genome research*, **31**, 1047-1059.
2. Park, D., Morris, A.R., Battenhouse, A. and Iyer, V.R. (2014) Simultaneous mapping of transcript ends at single-nucleotide resolution and identification of widespread promoter-associated non-coding RNA governed by TATA elements. *Nucleic acids research*, **42**, 3736-3749.
3. Robinson, M.D., Grigull, J., Mohammad, N. and Hughes, T.R. (2002) FunSpec: a web-based cluster interpreter for yeast. *BMC bioinformatics*, **3**, 35.
4. Kemmeren, P., Sameith, K., van de Pasch, L.A., Benschop, J.J., Lenstra, T.L., Margaritis, T., O'Duibhir, E., Apweiler, E., van Wageningen, S., Ko, C.W. *et al.* (2014) Large-scale genetic perturbations reveal regulatory networks and an abundance of gene-specific repressors. *Cell*, **157**, 740-752.
5. Choi, J.A. and Wyrick, J.J. (2017) RegulatorDB: a resource for the analysis of yeast transcriptional regulation. *Database (Oxford)*, **2017**.
6. Harbison, C.T., Gordon, D.B., Lee, T.I., Rinaldi, N.J., Macisaac, K.D., Danford, T.W., Hannett, N.M., Tagne, J.B., Reynolds, D.B., Yoo, J. *et al.* (2004) Transcriptional regulatory code of a eukaryotic genome. *Nature*, **431**, 99-104.
7. Morris, R.T., O'Connor, T.R. and Wyrick, J.J. (2010) Ceres: software for the integrated analysis of transcription factor binding sites and nucleosome positions in Saccharomyces cerevisiae. *Bioinformatics*, **26**, 168-174.
8. Hu, Z., Killion, P.J. and Iyer, V.R. (2007) Genetic reconstruction of a functional transcriptional regulatory network. *Nature genetics*, **39**, 683-687.
9. Reimand, J., Vaquerizas, J.M., Todd, A.E., Vilo, J. and Luscombe, N.M. (2010) Comprehensive reanalysis of transcription factor knockout expression data in Saccharomyces cerevisiae reveals many new targets. *Nucleic acids research*, **38**, 4768-4777.
10. Mao, P., Smerdon, M.J., Roberts, S.A. and Wyrick, J.J. (2016) Chromosomal landscape of UV damage formation and repair at single-nucleotide resolution. *Proceedings of the National Academy of Sciences of the United States of America*, **113**, 9057-9062.
11. Thorvaldsdottir, H., Robinson, J.T. and Mesirov, J.P. (2013) Integrative Genomics Viewer (IGV): high-performance genomics data visualization and exploration. *Brief Bioinform*, **14**, 178-192.
12. Rossi, M.J., Kuntala, P.K., Lai, W.K.M., Yamada, N., Badjatia, N., Mittal, C., Kuzu, G., Bocklund, K., Farrell, N.P., Blanda, T.R. *et al.* (2021) A high-resolution protein architecture of the budding yeast genome. *Nature*, **592**, 309-314.
13. Crooks, G.E., Hon, G., Chandonia, J.M. and Brenner, S.E. (2004) WebLogo: a sequence logo generator. *Genome research*, **14**, 1188-1190.
14. Holstege, F.C., Jennings, E.G., Wyrick, J.J., Lee, T.I., Hengartner, C.J., Green, M.R., Golub, T.R., Lander, E.S. and Young, R.A. (1998) Dissecting the regulatory circuitry of a eukaryotic genome. *Cell*, **95**, 717-728.
15. Consortium, E.P. (2012) An integrated encyclopedia of DNA elements in the human genome. *Nature*, **489**, 57-74.

16. Khurana, E., Fu, Y., Colonna, V., Mu, X.J., Kang, H.M., Lappalainen, T., Sboner, A., Lochovsky, L., Chen, J., Harmanci, A. *et al.* (2013) Integrative annotation of variants from 1092 humans: application to cancer genomics. *Science*, **342**, 1235587.
17. Roadmap Epigenomics, C., Kundaje, A., Meuleman, W., Ernst, J., Bilenky, M., Yen, A., Heravi-Moussavi, A., Kheradpour, P., Zhang, Z., Wang, J. *et al.* (2015) Integrative analysis of 111 reference human epigenomes. *Nature*, **518**, 317-330.
18. Sivapragasam, S., Stark, B., Albrecht, A.V., Bohm, K.A., Mao, P., Emehiser, R.G., Roberts, S.A., Hrdlicka, P.J., Poon, G.M.K. and Wyrick, J.J. (2021) CTCF binding modulates UV damage formation to promote mutation hot spots in melanoma. *The EMBO journal*, **40**, e107795.
19. Mao, P., Brown, A.J., Esaki, S., Lockwood, S., Poon, G.M.K., Smerdon, M.J., Roberts, S.A. and Wyrick, J.J. (2018) ETS transcription factors induce a unique UV damage signature that drives recurrent mutagenesis in melanoma. *Nature communications*, **9**, 2626.
